# Single-cell immunophenotyping identifies CD8^+^GZMK^+^IFNG^+^ T cells as a key immune population in cutaneous Lyme disease

**DOI:** 10.1172/jci.insight.196741

**Published:** 2026-02-23

**Authors:** Edel Aron, Hailong Meng, Alexia A. Belperron, Paraskevas Filippidis, Kenneth R. Dardick, Steven H. Kleinstein, Linda K. Bockenstedt

**Affiliations:** 1 Program in Computational Biology and Biomedical Informatics, Yale University, New Haven, Connecticut, USA.; 2 Department of Pathology, and; 3 Department of Internal Medicine, Yale School of Medicine, New Haven, Connecticut, USA.; 4 Mansfield Family Practice, Storrs, Connecticut, USA.; 5 Department of Immunobiology, Yale School of Medicine, New Haven, Connecticut, USA.

**Keywords:** Immunology, Infectious disease, Adaptive immunity, Skin, Transcriptomics

## Abstract

The skin lesion erythema migrans (EM) is the first clinical sign of Lyme disease, an infection due to the tick-transmitted bacterium *Borrelia burgdorferi* (*Bb*). Previously, we used scRNA-Seq to characterize the cutaneous immune response in the EM lesion, focusing on B cells. Here, with an expanded sample size, we profiled T cell responses in EM lesions compared to autologous uninvolved skin. In addition to CD4^+^ T cell subsets known to be abundant in the EM lesion, we identified clonally expanded CD8^+^GZMK^+^IFNG^+^ T cells that comprised cells with high or intermediate *IFNG* expression. These cells exhibited significant differential expression of IFN-regulated genes and included subsets with low cytotoxic gene expression, suggesting an inflammatory potential that may contribute to early defense against *Bb* within the EM lesion. In addition, we found that endothelial cells, fibroblasts, and pericytes were the main producers of key T cell–recruiting chemokines. These studies using single-cell transcriptomics with adaptive immune receptor sequencing provide a comprehensive interrogation of the cutaneous T cell response to *Bb* infection and insight into the orchestration of the skin barrier defense to this vector-borne pathogen.

## Abstract 2

We analyzed immune responses in Lyme disease-induced skin rashes and identified unique immune cell types that could play roles in defense or disease mechanisms.

## Graphical abstract

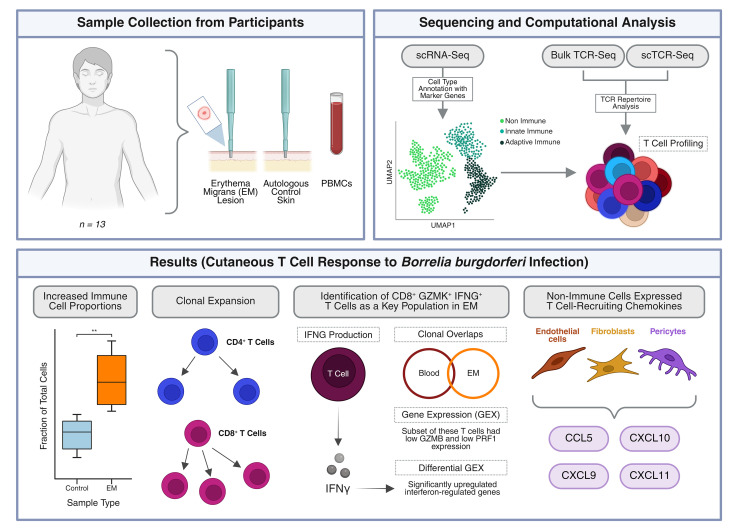

## Introduction

Lyme disease (LD), caused by infection with *Ixodes* tick-transmitted spirochetes of the *Borrelia burgdorferi sensu lato* (*Bb*) family (1), is the most common vector-borne disease in North America and Europe (2). An expanding erythematous skin lesion known as erythema migrans (EM) is the first clinical sign of infection in 70%–80% of patients and arises at the bite site within 1 month of infection. If unchecked at this barrier entry site, *Bb* may disseminate to cause neurologic, cardiac, and rheumatologic manifestations. The infection readily responds to antibiotics, although some people experience protracted symptoms and a minority develop postinfectious sequelae. These include persistence of inflammatory arthritis despite antibiotics (postinfectious Lyme arthritis) with autoimmune features that resolve with antirheumatic therapies (3, 4) and a posttreatment LD syndrome of cognitive dysfunction, musculoskeletal pain, and fatigue of unclear etiology (5–7). Strong evidence supports type II IFN responses contributing to disease pathogenesis from the earliest stages of infection when EM is present (8–10), as well as for dysregulation of such responses leading to postinfectious Lyme arthritis (11). Understanding the orchestration of host defense at the skin barrier site is a key factor in preventing LD and its adverse sequelae.

EM appears at a time when the actions of tick saliva on local skin immunity are estimated to wane (approximately 2 weeks after tick detachment) (12). At this stage, histopathology and flow cytometry studies of the lesion reveal that IFN-γ–producing T cells, monocytes/macrophages, and DCs comprise the main cells infiltrating dermal tissues, along with a variable presence of B cells and plasma cells (13–15). A transcriptomic microarray and pathway analysis revealed the dominance of an IFN signature, with gene expression of *IFNG* but not *IFNA* detected in the skin (8).

B cells are a critical host defense against *Bb* infection yet are underrepresented in normal skin in comparison with other cell types (16, 17). To further investigate the role of B cells in host defense at this barrier site, our group performed single-cell transcriptomics (scRNA-Seq) alongside B cell receptor and T cell receptor (TCR) sequencing to interrogate the cutaneous immune response in LD, with an emphasis on the B cell response (18). When comparing the composition of cells within EM lesions to those in autologous uninvolved skin, we found that the lesions were enriched for B cells along with plasma cells. EM B cells had clonal relatives in the blood, upregulated MHC class II genes, and exhibited a distinct signature for MHC class II antigen presentation and IFN-γ signaling. The presence of a large unmutated IgM memory cell population in the EM lesions supported an extrafollicular B cell response that may be critical for controlling spirochetes at this early stage of infection. A limited analysis of EM T cells revealed clones that could be traced to the circulation. These cells displayed gene signatures of cell trafficking and costimulation with CD28 as well as antigen-driven selection, as evidenced by clonal expansion. Given the prominence of T cells in the EM lesions, in this study, we sought to conduct a more comprehensive interrogation of the cutaneous T cell response in LD using an expanded sample size. Our results demonstrate involvement of a variety of T cell subsets, including a notable enrichment of CD8^+^GZMK^+^IFNG^+^ T cells, and through cytokine and chemokine analyses, offer insights into the role skin cells play in orchestrating host defense against this vector-borne pathogen.

## Results

### All defined T cell subsets are enriched in the EM lesion.

**Figure 1:**
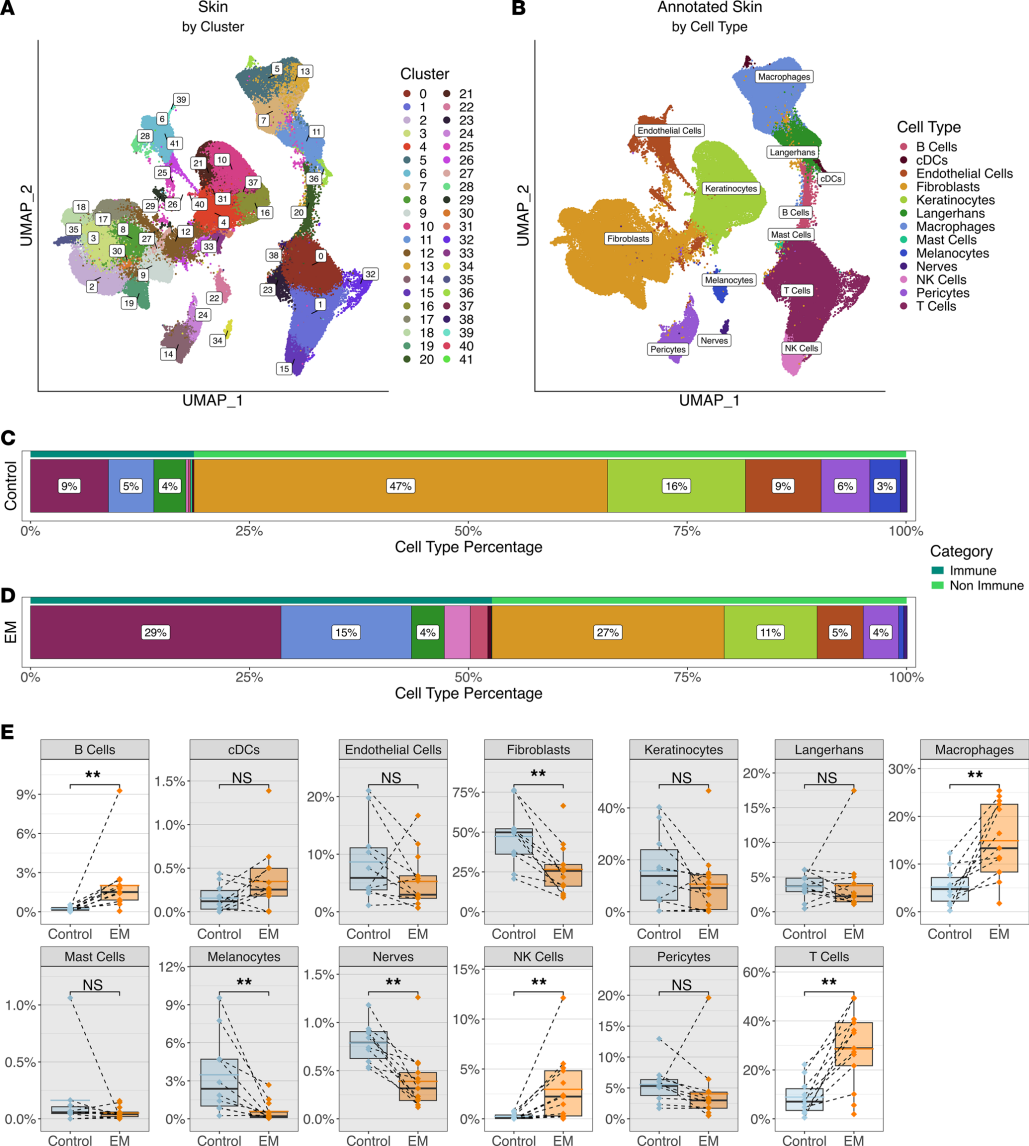
Clustering and annotation of the skin cells. (**A**) UMAP representing clustering of the combined control and EM skin data. At a resolution of 1.0, there were 42 clusters formed. (**B**) UMAP of skin cell types annotated using canonical marker genes. cDCs, cycling dendritic cells. (**C** and **D**) Proportions of cell types within the control and EM samples, respectively, grouped by immune (dark green bars) and nonimmune cell types (light green bars), then arranged in decreasing order by percentage. Proportions were averaged by participant for each cell type. Colors correspond to the cell types in **B**. (**E**) Comparison of the frequencies of each cell type between control and EM samples. Points represent results from individual participants and the dashed lines connect paired samples. The solid black horizontal lines within each box plot indicate the median and the blue or orange lines indicate the mean for each sample type and cell type across participants. Each box plot is bounded by the 25th and 75th percentiles (bottom and top lines respectively) of the data, with whiskers extending to the minimum and maximum values and additional points representing outliers. Gray backgrounds indicate cell types that did not have significant increases in EM compared to control samples. Significance was calculated using a paired Wilcoxon’s signed-rank test (**P < 0.01).

To characterize the cellular composition of the EM lesion, we used the 10x Genomics platform to perform scRNA-Seq along with adaptive immune receptor sequencing on cells from whole-skin digests of EM and uninvolved autologous (control) skin biopsies from 13 participants with acute LD (Table 1) (19). Analysis of the combined data from EM lesions and control skin samples for all participants identified 42 cell clusters (Figure 1A and Supplemental Figure 1A; supplemental material available online with this article; https://doi.org/10.1172/jci.insight.196741DS1). These clusters corresponded to 13 distinct cell types based on the expression of canonical marker genes (Figure 1B and Supplemental Figure 1B): B cells, cycling DCs, endothelial cells, fibroblasts, keratinocytes, Langerhans cells, macrophages, mast cells, melanocytes, NK cells, nerve cells, pericytes, and T cells.

As expected, the control skin samples were mainly composed of fibroblasts (47%) and keratinocytes (16%) (Figure 1C) (20). T cells (9%) dominated the immune cell population, with B cells (<1%) rarely detected, in line with previous studies (17, 18). The proportions of most immune cells in the EM lesion increased in comparison with control skin, with T cells remaining the largest cell population overall (29%) (Figure 1, C and D). Significant increases in the frequencies of individual immune cell types were found in the EM, including B cells, macrophages, NK cells, and T cells (Wilcoxon’s signed-rank test, *P* < 0.002), with corresponding decreases in frequencies of nonimmune cells (Figure 1E). Consequently, more than half (56%) of the cells in the EM lesions were immune related, as opposed to 21% in the control skin.

**Figure 2:**
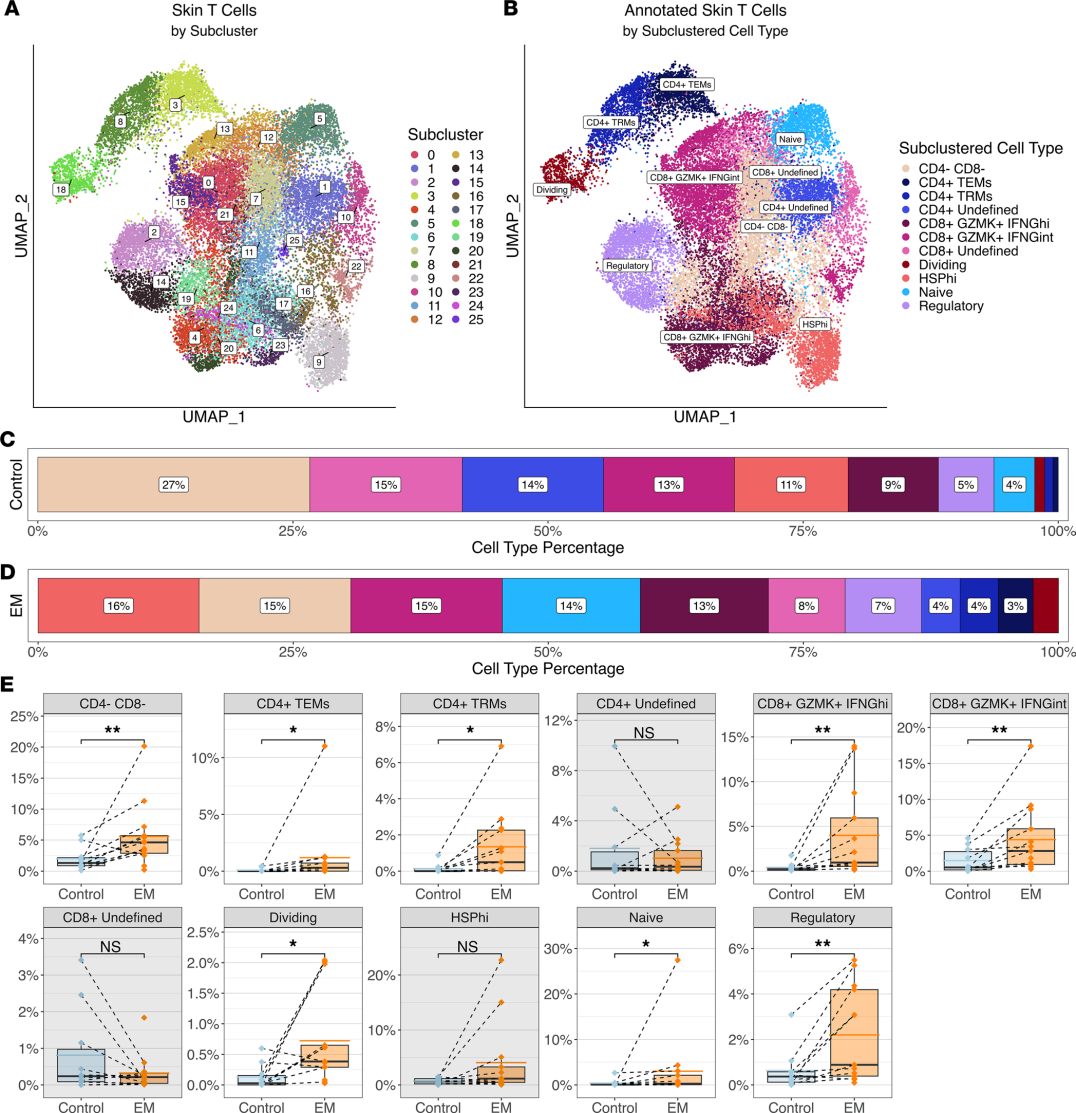
Clustering and annotation of the skin T cells. (**A**) UMAP representing clustering of the subclustered T cells from the combined control and EM skin data. At a resolution of 1.5, there were 26 clusters formed. (**B**) UMAP of skin T cell types annotated using canonical marker genes. (**C** and **D**) Proportions of cell types within the control and EM samples, respectively, arranged in decreasing order by percentage. Proportions were averaged by participant for each cell type. Colors correspond to the cell types in **B**. (**E**) Comparison of the frequencies of each cell type between control and EM samples. Cell frequencies are relative to all skin cells, not just the total T cells. Points represent results from individual participants and the dashed lines connect paired samples. The solid black horizontal lines within each box plot indicate the median and the blue or orange lines indicate the mean for each sample type and cell type across participants. Each box plot is bounded by the 25th and 75th percentiles (bottom and top lines respectively) of the data, with whiskers extending to the minimum and maximum values and additional points representing outliers. Gray backgrounds indicate the cell types that did not have significant increases in EM compared to control samples. Significance was calculated using a paired Wilcoxon’s signed-rank test (**P < 0.01, *P < 0.05).

T cells were further divided into 26 clusters (Figure 2A) and annotated using canonical marker genes into 11 distinct subsets (Figure 2B and Supplemental Figure 2). While we were able to assign clear labels to 9 of these subsets, 2 subsets of CD4^+^ and CD8^+^ T cells were designated as “undefined” due to the lack of additional gene markers or cell surface protein data that would permit further characterization. In addition to CD4^+^, CD8^+^, and FOXP3^+^ Treg cell subsets, the 9 identified T cell subsets included double-negative (CD4^–^CD8^–^) T cells, dividing T cells, naive T cells, and a group of cells that upregulated heat shock protein genes, designated as HSP^hi^ T cells (Figure 2B). While HSP^hi^ T cells may be stressed or dying cells that are often seen in scRNA-Seq data due to tissue processing (21), these cells could include a population experiencing cellular stressors in the context of skin infection. No NKT cells were identified. Even though the proportions of the T cell subsets varied between the EM and control skin cells (Figure 2, C and D), all defined T cell subsets (except for the HSP^hi^ T cells) were significantly increased in the EM when compared with controls (Figure 2E).

CD4^+^ T cells were subdivided into 2 groups representing CD4^+^ effector memory (Tem) and CD4^+^ tissue-resident memory (Trm) subsets, in addition to the “undefined” CD4^+^ T cell subset. The distinction between the CD4^+^ Tem and CD4^+^ Trm cell subsets was primarily based on expression of integrins and cytotoxic markers, as other distinguishing markers such as *CD69* were not detected in either subset. Characterization of the Tem cell subset revealed the presence of the tissue residency marker *CXCR6* and the memory T cell marker *KLRB1*. This subset also expressed the exhaustion marker *PDCD1* and the postactivation inhibitory molecule *CTLA4*, but not cytotoxic markers such as *GZMA* or tissue-homing markers such as *CCR7*. The CD4^+^ Trm cells also expressed *CXCR6*, *KLRB1*, and *PDCD1*, but could be distinguished from the Tem cells by their high expression of the signaling regulator *RGS1* and integrins such as *ITGAE* and *ITGA4* (as well as low expression of *CCR7*), suggesting an activated or cytotoxic phenotype. This subset also included activated T cells with the potential for Th1-like function as implied by expression of *GZMA*, *IFNG*, and the transcription factor *TBX21*.

We initially defined Tregs by their expression of *FOXP3* (8). FOXP3^+^ Tregs have been identified in peripheral blood and synovial fluid of people with Lyme arthritis, and a reduction in their numbers and ability to suppress IFN-γ production has been associated with immune dysregulation leading to postinfectious Lyme arthritis (22). FOXP3^+^ T cells can exhibit plasticity and, depending on the inflammatory environment, can secrete IFN-γ (23). In EM, the presence of immunosuppressive FOXP3^+^ T cells has been inferred by the detection of *IL10* mRNA in EM immune cells by in situ hybridization and IL-10 protein in blister fluid from EM lesions (13, 14). FOXP3^+^ T cells have been demonstrated in EM lesions by immunohistochemistry, although the cytokines they express within the tissue have not been directly assessed (8). We therefore characterized T cell subsets using differential gene expression of *IL10* in addition to *IFNG*, as FOXP3^+^ T cells can upregulate *IFNG* in the context of Th1-mediated immune responses and still retain their immunosuppressive potential (24, 25). Although multiple T cell subsets exhibited expression of *IL10*, FOXP3^+^ Tregs (which were not exclusively confined to the CD4^+^ subset) were not the main source of IL-10 as expected based on previous studies (22). Instead, the CD4^+^ Trm cells, which were FOXP3^–^, had the highest proportion of cells expressing *IL10* among all T cell types (Supplemental Figure 3, A–F). These CD4^+^FOXP3^–^IL10^+^ Trm cells are reminiscent of type I regulatory (Tr1) T cells, as they included cells that express *CTLA4*, *LAG3*, and *TGFB1* (as well as *ICOS*, *PDCD1*, and *TOX2*), but not *BCL6*, a transcription factor involved in germinal center formation (26–28). Tr1 T cells can regulate immune responses through gene expression as well as through induction of apoptosis (29–31). Accordingly, we found that *TNFSF10* was expressed within this population, suggesting that these T cells may suppress inflammation through apoptosis in addition to the secretion of IL-10 (Supplemental Figure 3, G–I).

*IFNG* expression was detected in FOXP3^+^ Tregs as well as multiple CD4^+^ T cell subsets, especially CD4^+^ Trm cells as predicted by their activation markers and expression of *TBX21* (Supplemental Figure 3, J–L). However, we unexpectedly found the greatest proportion of *IFNG* expression within the CD8^+^GZMK^+^ T cell population. This cluster was subdivided based on the level of *IFNG* expression into 2 groups, namely CD8^+^ GZMK^+^IFNG^hi^ T cells and CD8^+^GZMK^+^IFNG^int^ T cells. CD8^+^ T cells have been identified in EM lesions in prior studies and are considered to serve cytotoxic functions, although they have not been fully characterized (14, 32). To assess the cytotoxic potential of these cells, we analyzed the expression of *GZMB* and *PRF1* (perforin) and found that while these genes were expressed by cells within both populations, we observed a sizeable number of cells with very low or nondetectable levels of these cytotoxic genes (Supplemental Figure 4, A and B). The CD8^+^GZMK^+^IFNG^hi^ group had a larger proportion of GZMB^lo^PRF1^–^ cells in comparison with the CD8^+^GZMK^+^IFNG^int^ T cells (Supplemental Figure 4, C–F). These CD8^+^GZMB^lo^GZMK^+^IFNG^hi^PRF1^–^ cells were enriched in the EM lesion and are reminiscent of tissue-enriched GZMK^+^ T cells (T_teK_ cells) that were initially identified in rheumatoid synovium and subsequently found at barrier sites such as the lung, intestine, and skin (33).

The presence of cells expressing *GZMB* and *PRF1* within CD8^+^GZMK^+^IFNG^hi^ and CD8^+^GZMK^+^IFNG^int^ subclusters suggested that these populations could also contain terminal effector memory reexpressing *CD45RA* (Temra) cells, which have high cytotoxic potential (34). As we had no corresponding protein-based phenotyping data, we computationally inferred the presence of spliced *CD45* variants to identify a possible Temra subset (see Methods). We found that a large proportion of possible Temra cells were in the CD8^+^GZMK^+^IFNG^+^ subsets (Supplemental Figure 5). Interestingly, the IFNG^int^ T cells also contained a higher proportion of cells expressing the *CD45RB* isoform, which has been associated with virus-specific CD8^+^ memory T cells (35).

### CD8^+^GZMK^+^IFNG^hi^ T cells exhibit differential expression of genes distinct from CD8^+^GZMK^+^IFNG^int^ T cells in the EM lesion.

**Figure 3:**
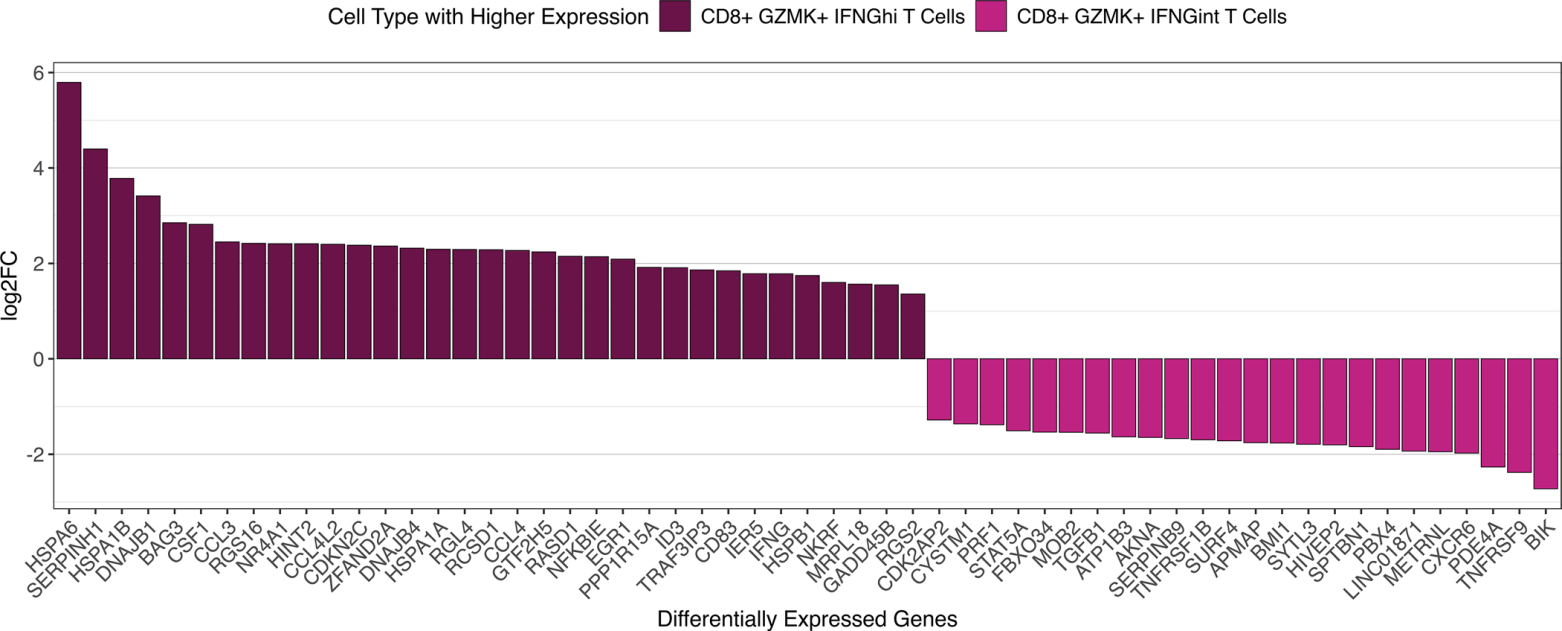
Differentially expressed genes between CD8^+^GZMK^+^IFNG^hi^ T cells and CD8^+^GZMK^+^IFNG^int^ T cells in the EM lesion only. Bar plot showing the top differentially expressed genes in EM CD8^+^GZMK^+^IFNG^hi^ T cells and CD8^+^GZMK^+^IFNG^int^ T cells with an adjusted *P* < 0.05, arranged by log_2_FC. Note that a negative log_2_FC reflects genes being expressed in the CD8^+^GZMK^+^IFNG^int^ T cells, not downregulation.

CD8^+^GZMK^+^IFNG^hi^ T cells may represent cells with similar differentiation trajectories as CD8^+^GZMK^+^IFNG^int^ T cells in the EM lesion. We ran a pseudobulk differential gene expression analysis comparing these 2 cell populations within the EM lesion to gain insight into which genes distinguish them other than the relative amount of *IFNG* expression (Figure 3). In addition to *IFNG*, CD8^+^GZMK^+^IFNG^hi^ T cells upregulated several *HSP* genes, including members of the HSP70 (*HSPA1B*, *HSPA1A*, *HSPA6*) and the HSP70-partner HSP40 (*DNAJB1*, *DNAJB4*) families, suggesting that they were exhibiting cell stress or even potentially inducing cytotoxicity (36–38). This population also included genes that promote (*CCL3*, *CCL4*, *CD83*) or modulate (*EGR1*, *NFKBIE*, *NKRF*, *TRAF3IP3*) inflammatory responses or affect T cell migration (*RGS2*, *RGS16*) (39–42). In contrast, CD8^+^GZMK^+^IFNG^int^ T cells upregulated genes involved with T cell activation and survival (*TNFRSF9*), cytotoxicity (*PRF1*), tissue localization (*CXCR6*), and regulation (*PDE4A*, *TGFB1*) (43). Thus, these cells may represent populations that evolve from common precursors yet achieve different functions depending on the skin microenvironment.

### EM T cells are clonally expanded, and a subset is shared with the blood.

**Figure 4:**
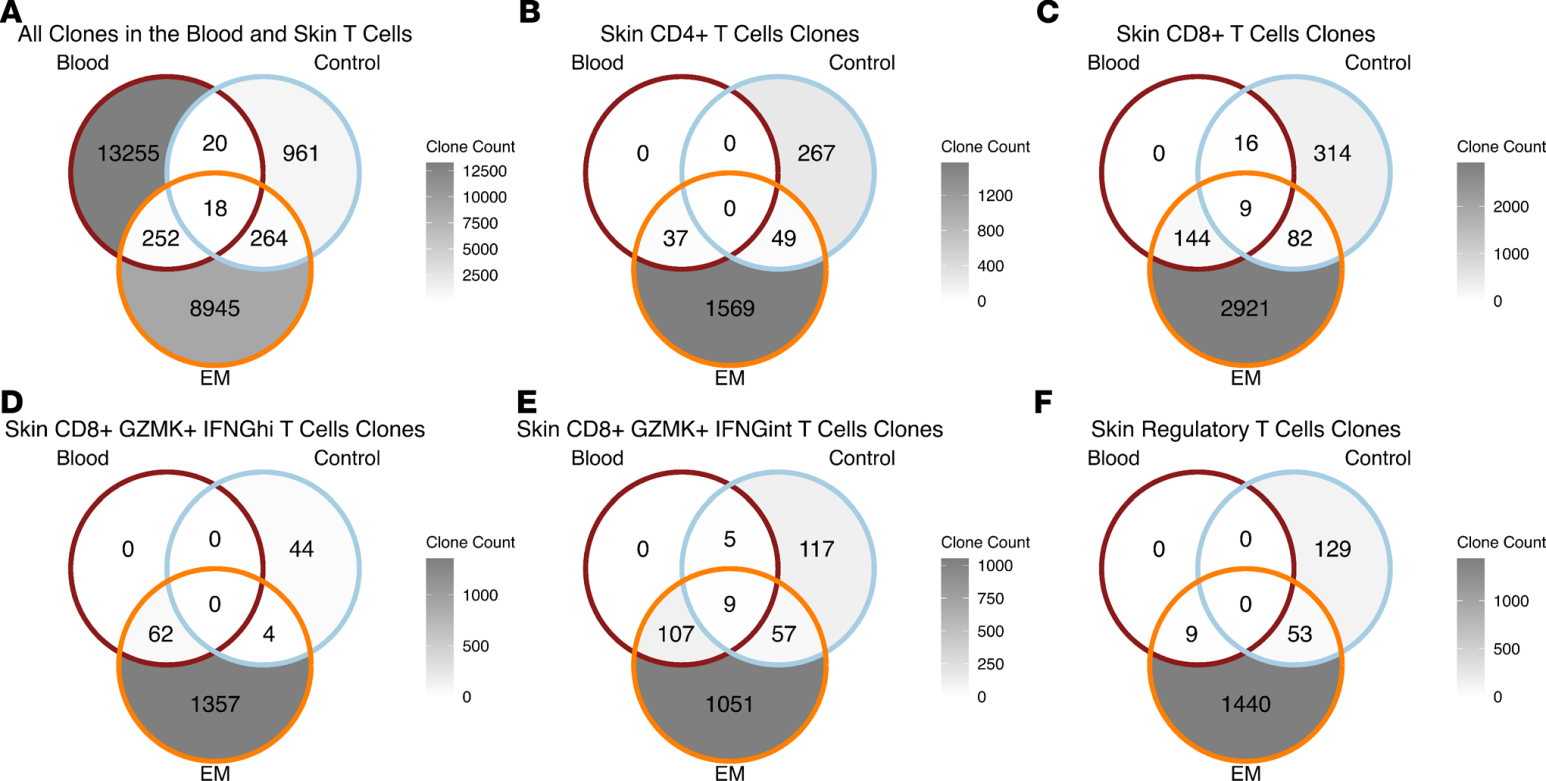
T cell clonal overlaps between the blood and the skin. (**A**) Clonal overlaps between all blood T cells (dark red) and skin T cells, with the latter split into EM (orange) and control (blue) skin. (**B** and **C**) Clonal overlaps between blood T cells and indicated skin CD4^+^ T cells (CD4^+^ effector memory T cells, CD4^+^ tissue-resident memory T cells, and CD4^+^ undefined T cells) or CD8^+^ T cells (CD8^+^GZMK^+^IFNG^hi^ T cells, CD8^+^GZMK^+^IFNG^int^ T cells, and CD8^+^ undefined T cells), respectively. (**D**–**F**) Clonal overlaps between the blood T cells and the skin CD8^+^GZMK^+^IFNG^hi^ T cells, CD8^+^GZMK^+^IFNG^int^ T cells, and Tregs, respectively. Values represent the number of unique TCRs identified per tissue. In **B**–**F**, counts for clonotypes only in blood T cells are zero, as only clonotypes for specified skin cell types are counted.

Immune repertoire analysis revealed that the increased T cell count in the EM as compared with the control skin results from both clonal expansion as well as an increase in the number of clonotypes (Supplemental Figure 6). To better understand this expansion and investigate potential migration between the skin and the blood, we quantified unique clonotypes across tissues (Figure 4 and Supplemental Figure 7). As expected, we observed that the blood T cells had the greatest number of clonotypes, but 96.5% of them were singletons (not expanded). Only 1.99% of these T cell clonotypes were shared with the EM lesion and 0.28% with the control skin (Figure 4A). A 2-sample proportion test revealed that the overlap between the blood and the EM clonotypes was significantly more than between the blood and the control skin clonotypes (*P* = 0.004).

We determined which T cell subsets within the EM lesion had clonotypes shared with the blood and were distinct from those in control skin. A minority of the total EM CD4^+^ T cell clonotypes (2.24%) and none of the control skin clonotypes were traced to the circulation (*P* = 0.014) (Figure 4B), in comparison with 5.94% of EM and 4.85% of control skin CD8^+^ T cell clonotypes (*P* = 0.017) (Figure 4C). When comparing CD8^+^GZMK^+^ T cells, a greater percentage of EM IFNG^int^ T cell clonotypes (9.48%) than EM IFNG^hi^ T cell clonotypes (4.36%) shared TCRs with cells in the blood (Figure 4, D and E). Moreover, both EM and control skin CD8^+^GZMK^+^IFNG^int^ T cell clonotypes were detected in the blood (Figure 4E), with the overlap between the blood and the EM being significantly greater than the blood and the control (*P* = 0.048). In contrast, none of the control skin CD8^+^GZMK^+^IFNG^hi^ T cell clonotypes were detected in the blood. We also examined the Treg subset and found only EM Treg clonotypes in the blood, similar to the pattern seen with CD4^+^ and CD8^+^GZMK^+^IFNG^hi^ T cell clonotypes (Figure 4F).

### Nonimmune cells express T cell–recruiting chemokines in EM lesions.

**Figure 5:**
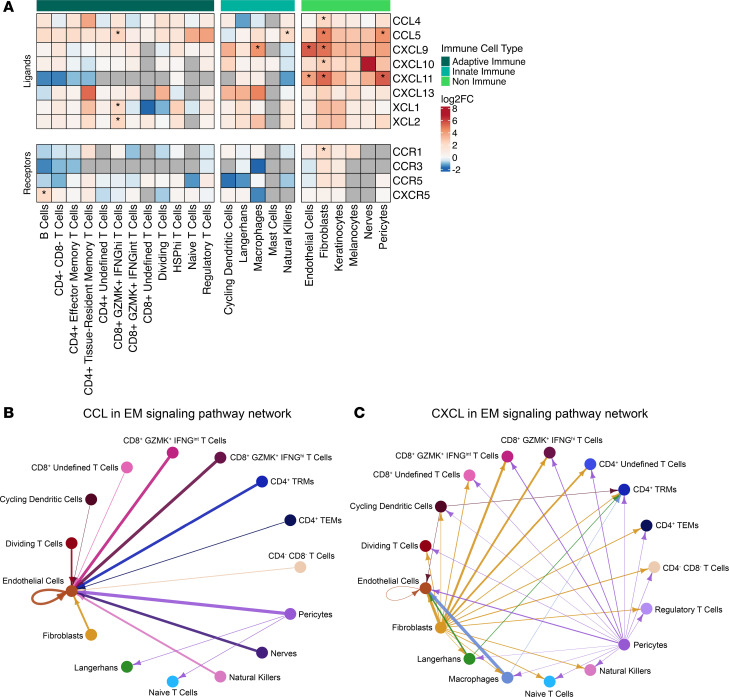
Gene expression of chemokine ligand and receptor genes in the skin. (**A**) Heatmap showing the average fold change of the expression of select chemokine-related genes across skin cell types. The bar on the top designates cell types as immune- or nonimmune-related (dark green for adaptive immune cell types, cyan for innate immune cell types, and bright green for nonimmune cell types). Within the heatmap, red indicates upregulation, and blue downregulation, with asterisks denoting statistically significant changes (FDR < 0.05). Gray cells, if present, indicate that gene was filtered out by DESeq2 for that cell type. Differential expression was calculated between the EM and control portions of each cell type. (**B** and **C**) Circle plots showing the inferred communication networks within the EM for the CCL (**B**) and CXCL (**C**) pathways. Arrows point from the sender cells to the receiver cells, with edges colored by sender. Edge width increases as the signal strength increases. Cell types without any inferred interactions are not shown.

We next examined which skin cell types might be responsible for recruiting the T cells to the EM. Prior studies reported that DCs and macrophages were the principal immune cells attracting T cells to sites of *Bb* infection in tissues (8, 44). However, dermal fibroblasts are also important sources of lymphocyte-attracting cytokines and chemokines, as has been demonstrated in studies of autoimmune and inflammatory skin diseases (45–47). Thus, we assessed chemokine expression in both immune and nonimmune cell subsets to identify cell types that may attract T cells and other immune cells to the EM lesion. We used differential gene expression analysis to compare the expression of all chemokine ligand and receptor genes in each cell type in the EM and the control skin (Figure 5A and Supplemental Figure 8). Several chemokine ligand and receptor genes were significantly expressed across a variety of cell types, with many nonimmune cell types differentially expressing at least 1 gene. We found significant upregulation of genes in nonimmune cells (endothelial cells, fibroblasts, and pericytes) as well as immune cells (B cells, CD8^+^GZMK^+^IFNG^hi^ T cells, macrophages, NK cells). In contrast, Langerhans cells and macrophages were the only cells exhibiting significant downregulation of chemokines, predominantly those that recruit neutrophils (*CXCL1*, *CXCL2*, *CXCL3*, *CXCL5*, and *CXCL8*), consistent with the paucity of neutrophils found on EM histopathology (48).

Fibroblasts significantly upregulated the greatest number of chemokine genes, including the T cell–recruiting chemokine *CCL5* (49) and its receptor *CCR1*, lymphocyte chemoattractants *CXCL9*, *CXCL10*, and *CXCL11*, as well as proinflammatory macrophage attractants *CCL4*, *CCL18*, *CCL19*, *CCL26*, and the neutrophil attractant *CX3CL1*. Significantly upregulated lymphocyte recruiting chemokine genes were also found in endothelial cells (*CXCL9* and *CXCL11*) and pericytes (*CXCL11*), but macrophages only upregulated *CXCL9*. The only significantly differentially expressed chemokine genes by T cells were found in the CD8^+^GZMK^+^IFNG^hi^ subset and included *XCL1* and *XCL2*, which recruit conventional DCs important for cross-presentation of antigen (50). In addition, this T cell subset significantly upregulated the T cell chemoattractant *CCL5*. Overall, most chemokines specifically involved with T cell recruitment were found to be upregulated in nonimmune cells.

To gain additional insight into the interactions between nonimmune skin cell types and T cell subsets, we examined predicted relationships mediated by CCL and CXCL chemokine signaling using CellChat. T cells, especially CD8^+^GZMK^+^ T cell subsets and CD4^+^ Trm cells, interacted primarily with endothelial cells via CCLs (Figure 5B), whereas fibroblasts interacted with numerous T cell subsets via CXCLs, with the strongest interactions with CD8^+^GZMK^+^ T cells (Figure 5C). Interestingly, when examining differential interaction strength among all cell types in the skin (Supplemental Figure 9), fibroblasts were predicted to have stronger interactions with CD8^+^GZMK^+^IFNG^hi^ T cells and weaker interactions with CD8^+^GZMK^+^IFNG^int^ T cells and CD4^+^ Trm cells. B cells were predicted to have the strongest interactions with CD8^+^GZMK^+^IFNG^int^ T cells in comparison with all other cell types. Macrophages, on the other hand, were predicted to interact strongly with CD4^+^ Trm cells and CD8^+^GZMK^+^IFNG^hi^ T cells, with weaker interactions with B cells and endothelial cells. These findings support contributions of nonimmune cells to the evolving T cell response in the EM lesion.

### IFN response pathways are enriched in the EM lesion, but only IFNG expression is significantly upregulated.

**Figure 6:**
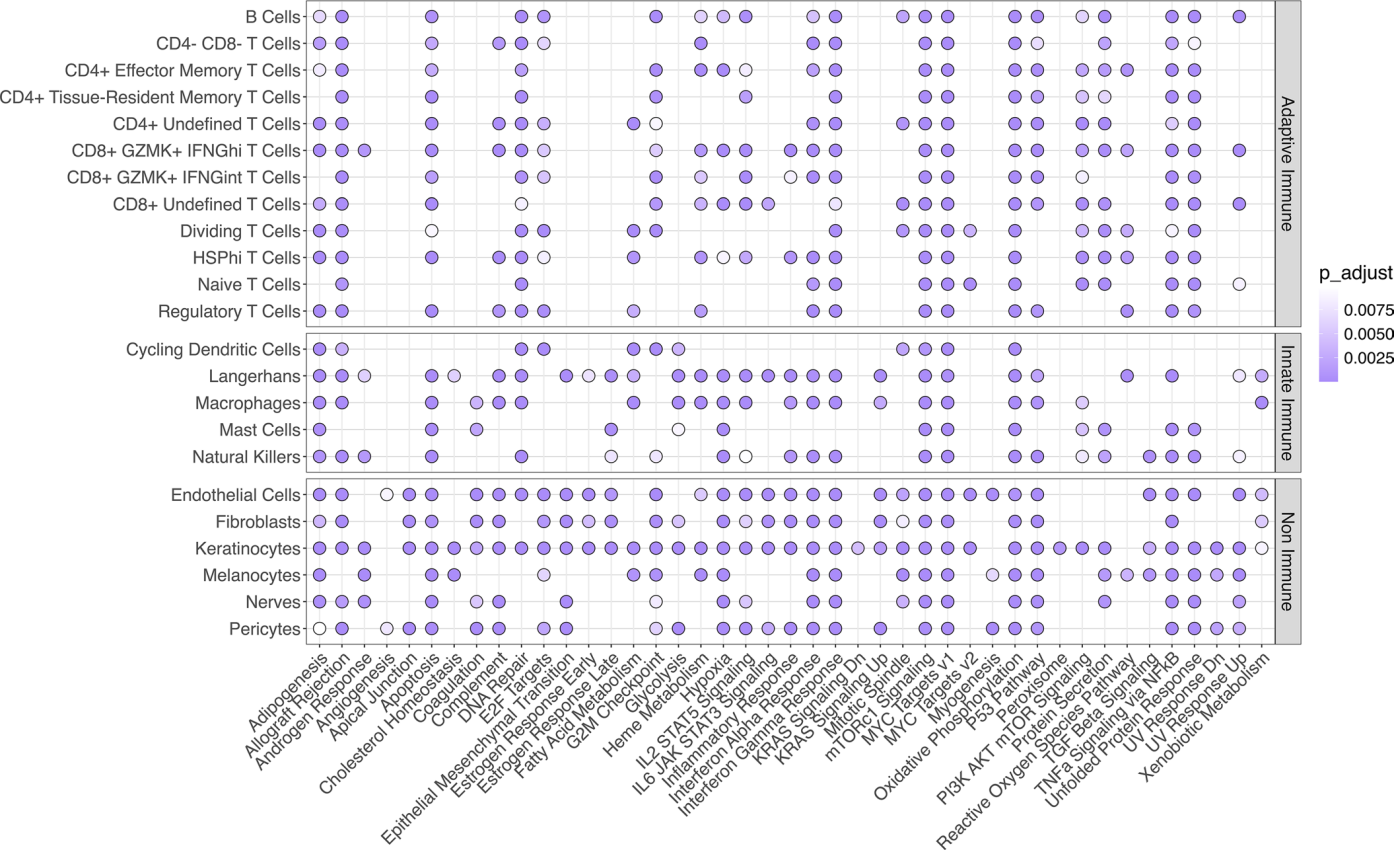
GSEA significance analysis using the MSigDB Hallmark database in the skin. Purple circles indicate significance (Bonferroni-corrected *P* < 0.01). Only pathways with significance in at least one cell type are shown.

In a previous study, IFN signaling (including the upstream regulators *IFNA* and *IFNG*) was implicated by pathway analysis of bulk EM transcriptomes, with only *IFNG* expression detected in the lesion (8). Since many of the chemokines we observed to be upregulated in the EM lesion are induced by IFNs, we sought to gain a better understanding of the biological pathways involved by performing gene set enrichment analysis (GSEA), comparing EM and control skin across cell types using the Human Molecular Signatures Database’s Hallmark reference. GSEA showed a total of 43 immune pathways (out of 50 total Hallmark pathways) with positive enrichment in at least one cell type, including pathways involving apoptosis, complement, interleukins, JAK/STAT signaling, IFN responses, TGF-β signaling, and TNF-α signaling (Figure 6). Both the IFN-α and the IFN-γ response pathways were significantly enriched in almost all cell types, as was the TNF-α signaling via the NF-κB pathway (except in a couple of innate immune cell populations).

**Figure 7:**
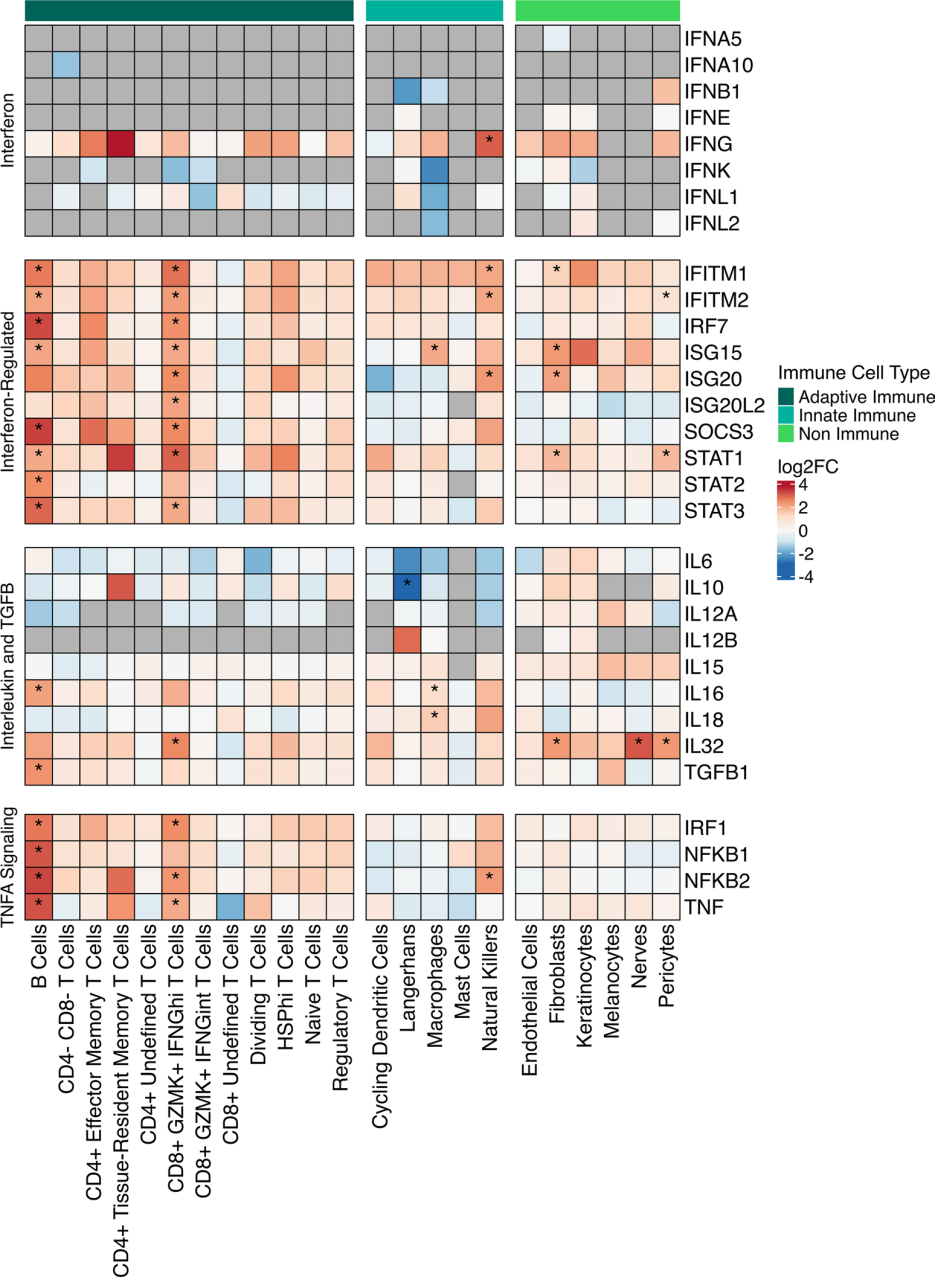
Gene expression of IFN, IFN-regulated, interleukin, and signaling-related genes in the skin. Heatmap showing the average fold change of the expression of select genes across skin cell types. The bar on the top designates cell types as immune- or nonimmune-related (dark green for adaptive immune cell types, cyan for innate immune cell types, and bright green for nonimmune cell types). Within the heatmap, red indicates upregulation, and blue downregulation, with asterisks denoting statistically significant changes (FDR < 0.05). Gray cells, if present, indicate that gene was filtered out by DESeq2 for that cell type. Differential expression was calculated between EM and control for each cell type.

To determine which IFNs might be produced locally in the skin and subsequently drive the IFN response signature, we carried out a differential gene expression analysis of EM versus control skin in every cell subset (Figure 7 and Supplemental Figure 10). Out of all the IFN genes, only *IFNG* was significantly upregulated (and only in NK cells); most other cell types exhibited increased *IFNG* expression, but at levels that did not achieve statistical significance. The only type I IFNs that were detected as having increased expression (albeit not significantly) were *IFNB1* in pericytes, *IFNE* in fibroblasts, keratinocytes, Langerhans cells, and pericytes, and *IFNK* in fibroblasts. When examining cells impacted by IFN signaling, we found that several IFN-regulated genes were significantly upregulated within the CD8^+^GZMK^+^IFNG^hi^ T cells, including *IFITM1*, *IFITM2*, *IRF1*, *IRF7*, *ISG20*, and *STAT1*. Most of these genes were also significantly upregulated within B cells, along with genes associated with IFN signaling and downstream effects of IFN.

### Select cytokine expression is upregulated in EM lesions.

We also investigated the expression of interleukin genes to identify which cells in the EM contribute to cell recruitment through cytokine production (Figure 7 and Supplemental Figure 11). GSEA suggested involvement of the IL-6/JAK/STAT3, TGF-β, and TNF-α signaling pathways. Although we did not detect significant changes in *IL6* in any cell type, we did see upregulation of *JAK1*, *STAT3*, *TGFB1*, and *TNF* in the B cells, with *STAT3* and *TNF* also upregulated in the CD8^+^GZMK^+^IFNG^hi^ T cells. Genes involved in downstream signaling of TNF-α such as *NFKB* and *STAT1* were upregulated in the B cells and CD8^+^GZMK^+^IFNG^hi^ T cells. Macrophages significantly upregulated *IL16* and *IL18*, which are known to induce *IFNG* expression and are consistent with an M1 phenotype (51). IL-16, which is a proinflammatory cytokine that also can serve as a chemoattractant and regulator of CD4^+^ T cell responses (52), was significantly upregulated in B cells as well. None of the T cell subsets showed increased expression of any interleukin genes, except for CD8^+^GZMK^+^IFNG^hi^ T cells that upregulated *IL32*, a cytokine that can promote FOXP3^+^ Treg cell development and influence DC function to promote proinflammatory T cell responses (53, 54). *IL32* was also significantly upregulated within the fibroblasts, nerve cells, and pericytes. These findings suggest that several cell types express key interleukins to help drive IFN production and modulate the recruitment and function of immune cells.

### Characterization of the inflammatory state of skin immune cells through metabolic profiling of gene expression.

Finally, we examined differential expression of genes in the Hallmark pathways that involve metabolism to gain insight into how T cells generate energy in the context of the EM lesion. Pathways examined were adipogenesis, bile acid metabolism, cholesterol homeostasis, fatty acid metabolism, glycolysis, heme metabolism, oxidative phosphorylation, peroxisome, and xenobiotic metabolism. We focused on oxidative phosphorylation and glycolytic pathways because of their importance in energy utilization in immune responses (Supplemental Figure 12).

Consistent with their acute activation, the CD8^+^GZMK^+^IFNG^hi^ T cells upregulated numerous genes in the oxidative phosphorylation pathway involved in mitochondrial ATP synthesis, essential for generation of energy required for T cell proliferation and effector function. These genes encode components of the respiratory chain complexes I (*NDUF* genes), II (*SDH* genes), III (*UQCR* genes), IV (*COX* genes), and V (*ATP5* genes) of the mitochondrial transport chain. CD8^+^GZMK^+^IFNG^hi^ T cells also upregulated many genes involved with glycolysis such as *ENO1*, which catalyzes the generation of pyruvate, and *LDH*, which converts pyruvate to lactate, promoting aerobic glycolysis (the Warburg effect). Numerous other upregulated genes regulate oxidative stress and protect cells from apoptosis and ferroptosis. For example, *LGALS1* serves as an autocrine negative regulator to prevent excessive inflammation, as does *BAX,* which typically coincides with the elimination of excess effector CD8^+^ T cells in the contraction phase of an immune response. Upregulation of *IDH2* in the CD8^+^GZMK^+^IFNG^hi^ T cells suggests that some cells may be entering a terminally differentiated state. Several metabolism-related genes were also significantly differentially expressed by macrophages. These included *HK2*, *LDH2*, and *PYGB*, which are associated with glycolysis (a trait of M1 macrophage differentiation) as well as respiratory chain complex genes involved in oxidative phosphorylation (a trait of M2 macrophage differentiation) (55). Taken together, these findings are consistent with cells in a variety of activation states within the EM samples and reflect the importance of an individual cell’s microenvironment to its evolving phenotype.

## Discussion

The skin represents the first barrier to infection by arthropod-transmitted pathogens and is the site where *Bb* spirochetes must establish infection and persist to complete their life cycles (56). Transmission of *Bb* to the mammalian host is preceded by replication, programmatic changes in gene expression, and migration from the *Ixodes* tick midgut to the salivary glands prior to deposition into the dermal layer of the skin at the bite site. Tick salivary protein interactions with newly expressed *Bb* proteins and the pharmacologic properties of tick saliva inhibit local immunity to permit *Bb* to successfully establish infection in the dermis (57). These actions of tick saliva as well as the introduction of spirochetes into the skin are likely to perturb the bite site microbiome (57). The EM lesion temporally appears after tick detachment when the local effects of tick saliva are expected to wane, and *Bb* (which is an extracellular pathogen) begins to migrate outward through the collagen-rich dermal connective tissue. By the time EM is brought to medical attention, the lesion histopathology reveals the dominance of DCs, macrophages, and T cells as the principal immune cells responding to *Bb* and to potential disruptions of the complex microbial community in the skin (32).

Earlier studies have characterized the immune cell infiltrates in EM lesions by in situ hybridization techniques (13), flow cytometry of cells along with cytokine analysis of fluid extracted from EM lesions (14), and through transcriptomic analysis of EM skin biopsies (8, 18). These techniques confirm the predominance of T cells and macrophages along with the elevated levels of inflammatory (*IFNG*, *IL1B*, *IL6*, *TNFA*) and antiinflammatory (*IL10*) cytokines in EM lesions. Phenotypic analysis of the T cell subsets from fluid in blisters experimentally induced over the EM lesion revealed that the proportion of CD4^+^ T cells (68%) was much higher than CD8^+^ T cells (20%), with the CD4^+^ T cell population dominated by CD27^–^CD45RO^+^ Tem cells and strongly polarized toward a Th1 phenotype (14). A transcriptomic analysis via microarray on whole-skin digests of the EM lesion supported some of these findings and also revealed a potential role for the kynurenine pathway and tryptophan metabolites in the regulation of the inflammatory response (8). A model for the cutaneous immune response based on transcriptomic microarray analysis has been proposed, supporting the viewpoint that innate immune cells (such as DCs and macrophages) responding to *Bb* inflammatory components recruit CD4^+^ Th1 cells that result in the strong IFN-γ signatures seen in the EM lesion (8).

Our studies using scRNA-Seq combined with scTCR-Seq on whole EM skin cell digests have provided information that refines our understanding of the host response to *Bb* in the skin. Our results reveal enrichment of many immune cell subsets in the EM lesion along with the expected dominance of T cells. CD8^+^ T cells constituted the largest T cell population, with both CD8^+^GZMK^+^IFNG^hi^ and CD8^+^GZMK^+^IFNG^int^ T cell subsets enriched in the EM lesion. Our analysis of *CD45* gene splicing also detected possible Temra cells mainly within the CD8^+^GZMK^+^IFNG^int^ T cell subset. Temra cells can have effector functions such as cytotoxicity and are associated with cell senescence in aging (58, 59); however, we observed an increased proportion of CD8^+^GZMK^+^IFNG^+^ T cells in EM lesions from all study participants (age range 31–76, median 68), indicating that their presence was not a function of aging per se.

Furthermore, we found that not all CD8^+^GZMK^+^IFNG^+^ cells expressed characteristic cytotoxic genes. A greater proportion of the CD8^+^GZMK^+^IFNG^hi^ T cells contained cells with low or undetectable levels of *GZMB* and *PRF1*, in comparison with the CD8^+^GZMK^+^IFNG^int^ T cells. These CD8^+^GZMB^lo^PRF1^–^ T cells are reminiscent of CD8^+^ T_teK_ cells found in rheumatoid arthritis synovium and at barrier sites, where they have been proposed to initiate or sustain inflammatory responses (33, 60). CD8^+^GZMK^+^ T cells can be activated in an antigen-independent fashion in response to cytokines alone and in this way share features of innate immune cells (33, 61). Although they are enriched in peripheral nonlymphoid tissues, they can also be detected in the circulation (61). Recently, GZMK was shown to activate the complement cascade in a proposed fourth pathway distinct from the classical, alternative, and lectin pathways (62). In that study, fibroblasts were the main source of complement components for GZMK-initiated complement activation. In our data, fibroblasts and macrophages in both EM and control skin have the highest average expression of complement genes. Taken together with the abundance of CD8^+^GZMK^+^ T cells in the EM lesion, it is possible that T cell production of GZMK activates complement through this fourth pathway.

We found clonal expansion of both CD8^+^GZMK^+^IFNG^hi^ T cells and CD8^+^GZMK^+^IFNG^int^ T cells in the EM lesion, with some clones expressing TCRs clonally related to T cells in the blood. Notably, some clonotypes were also shared between the 2 CD8^+^GZMK^+^IFNG^+^ T cell populations in the EM lesions. CD8^+^GZMK^+^IFNG^hi^ T cells in the control samples, however, do not share any clonotypes with blood T cells, in contrast with CD8^+^GZMK^+^IFNG^int^ T cells, in which a small number of clonotypes in the control skin were shared with the blood and/or the EM lesions. The 2 populations of CD8^+^GZMK^+^IFNG^+^ T cells may be related to one another in both the EM lesions and the blood, but only CD8^+^GZMK^+^IFNG^int^ cells from the blood and EM lesions have clonal relatives in the control skin. A comparison of the genes differentially expressed by CD8^+^GZMK^+^IFNG^hi^ T cells versus the CD8^+^GZMK^+^IFNG^int^ T cells revealed substantial differences, however, with the former including genes associated with inflammation and its regulation and the latter including genes associated with cytotoxicity. The kinetics of the responses of these subsets are not known, but together they may represent cells that respond to disruption of the skin barrier and introduction of *Bb* while maintaining a cytotoxic population that, along with NK cells, keeps the cutaneous microbiome in check.

Our GSEA identified both type I and type II IFN pathways, along with other immune pathways involved in host defense and immune regulation as significantly upregulated in the EM lesion. The finding that B cells and CD8^+^GZMK^+^IFNG^hi^ T cells exhibited robust differential expression of IFN-stimulated genes suggests important roles for both cell types in the early host defense and the *Bb*-specific CD4^+^ T cell responses. B cells upregulated *IL16* that promotes CD4^+^ Th1 cell differentiation, whereas CD8^+^GZMK^+^IFNG^hi^ T cells upregulated *IL32*, a cytokine important for immune responses against viruses and intracellular bacteria (51, 53, 54). IFN-γ produced by NK cells and CD8^+^GZMK^+^IFNG^hi^ T cells could drive fibroblast differentiation into inflammatory phenotypes. In this regard, we noted that fibroblasts, not macrophages, upregulated the broadest array of T cell–recruiting chemokines. Fibroblasts are the major component of the dermis and comprise diverse subtypes, including those involved in homeostasis of the extracellular matrix and others that have a role in immune responses, inflammation, and host defense against pathogens (63). Analyses of gene expression using microarrays have documented that primary human dermal fibroblasts exposed to *Bb* spirochetes upregulate expression of several cytokines and chemokines, including *IL6*, *IL8*, *CCL2*, *CCL5*, *CXCL1*, *CXCL2*, *CXCL6*, *CXCL9*, and *CXCL10* (64). These results have led to the hypothesis that dermal fibroblasts responding to the presence of *Bb* in the skin play a key role in recruiting immune cells to the infection site. Our study provides support for this hypothesis by showing the significant increase in gene expression of *CCL4*, *CCL5*, *CCR1*, *CCR5*, *CXCL9*, *CXC10*, and *CXC11* by fibroblasts in EM lesions. In addition, we found upregulation of chemokine genes by other nonimmune cells such as endothelial cells and pericytes, which may serve to recruit T cells and macrophages to perivascular tissues, consistent with the histopathology characteristic of EM lesions. A cell-cell communication tool predicted interactions of fibroblasts with T cell subsets via CXCL chemokines, as well as interactions of T cell subsets with endothelial cells via CCL chemokines. Based on the prediction of strengths of interactions among all cell types, we speculate that fibroblasts and macrophages may interact preferentially with distinct B and T cell populations within the EM lesion.

Fibroblasts also contribute to regulation of the inflammatory response and resolution of disease (63). We found that fibroblasts were the only skin cell type that exhibited significant differential expression of *IDO1* (Supplemental Figure 10), the gene for a rate-limiting enzyme for tryptophan catabolism to kynurenine (65) that was previously found to be upregulated in transcriptomic microarray analyses of EM digests (8). Tryptophan depletion can suppress T cell proliferation, and genes in the kynurenine pathway can induce apoptosis of Tregs and other T cells through activation of the aryl hydrocarbon receptor (66). *KMO* and *KYNU*, two other genes associated with tryptophan catabolism, were differentially expressed within the B cell population, suggesting activation of the kynurenine pathway. Kynurenine activation could potentially lead to apoptosis of B cells (67), thereby interfering with the B cell defense in the skin.

Our studies identifying nonimmune cells expressing genes associated with T cell recruitment to the site of *Bb* infection in the skin also show involvement of DCs and macrophages. Indeed, our CellChat analyses show the complexity of cell-cell interactions within the EM lesion, predicting T cell interactions with both stromal and immune cells such as macrophages. At other sites of *Bb* infection in humans, such as the heart, perivascular lymphohistiocytic infiltrates and plasma cells are seen, along with increased collagen production known to be a feature of the normal fibroblast response to cardiac injury (68). In Lyme neuroborreliosis, marked microglia proliferation is seen in the meninges of autopsy specimens (69), and both CD4^+^ and CD8^+^ T cells are found in the cerebrospinal fluid (CSF) (70). In vitro studies demonstrate that astrocytes, which are part of the stroma, respond with cytokine and chemokine production when exposed to *Bb*. These cells are known to be important in host defense and tissue repair, similar to fibroblasts (71). In Lyme arthritis, synovial fibroblasts produce inflammatory cytokines and chemokines in response to infection and are thought to contribute to the perpetuation of inflammation in postinfectious Lyme arthritis (72, 73).

While the immune changes we observe in the EM lesion may be triggered by *Bb* infection, they may also reflect, in part, ongoing immune responses resulting from tick feeding and disruption of the skin microbiome. European studies that examined the cutaneous immune response to naturally acquired bites from *Ixodes ricinus* ticks that did not harbor *Borrelia* *spp*. spirochetes have documented increasing presence of lymphocytes over time (12, 74). Flow cytometry analyses identified increases in both B and T cells, with a decrease in the CD4^+^ to CD8^+^ T cell ratio in comparison to uninvolved skin, and an increase in the Trm cells. While our skin samples were taken at the leading edge of the EM lesion (away from the bite site), some of the changes we observe in the immune cell populations could result from tick feeding and changes in the microbiome (74).

There is limited information on the role of CD8^+^ T cells in LD in humans, and no prior study has phenotypically or functionally characterized this population in the EM lesion. A European study examining TCR β chain usage of T cells isolated from CSF and peripheral blood of people presenting with neuroborreliosis identified clonal expansion of CD8^+^ T cells within the CSF that had a memory phenotype (CD28^+^CD45RO^+^) and which decreased in number after treatment (70). These cells expressed *CCR5* and *CD69*, produced IFN-γ after in vitro stimulation with anti-CD3/anti-CD28 beads, and released TNF-α and to a lesser extent IFN-γ when incubated with DCs and *Bb* lysate. They did not exhibit cytotoxicity, however, when stimulated with EBV-transformed B cells pulsed with *Bb* lysate. One proposed interpretation of these findings is that the CSF CD8^+^ T cell clonotypes helped to initiate the inflammatory response to *Bb* infection in the CNS without exerting cytotoxic effects on antigen-presenting cells that may be activating them. Other studies have identified expansion of cytotoxic CD8^+^ T cells in blood samples from people experiencing protracted symptoms after neuroborreliosis when their PBMCs were cocultured with macrophages pre-exposed to live spirochetes (75). *Bb* antigen–specific cytotoxicity has also been observed in CD8^+^ T cell lines derived from peripheral blood and synovial fluid of people with Lyme arthritis (76). A more recent study found a greater percentage of CD8^+^ T cells in the peripheral blood of people who had postinfectious Lyme arthritis in comparison with those who resolved joint inflammation with antibiotic therapy (77). In contrast with rheumatoid arthritis, in which the CD8^+^ T cells had low levels of GZMB and PRF1 (33), CD8^+^ T cells found in Lyme arthritis samples had elevated levels of these cytotoxic molecules. Given the prevalence of CD8^+^ T cells near vasculature on immunohistochemistry, it was postulated that cytotoxic T cells may lead to microvascular changes that are characteristic of postinfectious Lyme arthritis. Taken together with our results identifying CD8^+^GZMK^+^IFNG^+^ T cell subsets with low cytotoxic gene expression, CD8^+^ T cells in LD may comprise distinct subsets, one capable of promoting an inflammatory state early in *Bb* infection in the skin or CNS (while minimizing tissue damage) and others with cytotoxic potential contributing to adverse sequelae.

In summary, our single-cell analysis identified CD8^+^GZMK^+^IFNG^+^ T cells enriched in the EM lesion that may play an important role in the immune response at the skin barrier site to *Ixodes* tick–transmitted *Bb* infection. We postulate that CD8^+^GZMK^+^IFNG^+^ T cells (along with B cells) instructed by nonimmune cells such as fibroblasts shape the cellular response to the introduction of *Bb* and perturbation of the cutaneous microbiome. Although we have focused on the tick-transmitted bacterium *Bb*, our findings may have relevance to the cutaneous host defense against other vector-borne pathogens. Conclusions may be limited by the relatively small number of participants (with skewing toward older individuals and women), as well as limitations in the ability of single cell transcriptomics to detect low-abundance transcripts. Additional studies to assess specific interactions among immune and nonimmune cells, such as spatial transcriptomics and protein expression analyses, could further enhance our understanding of the initial host defense against vector-borne pathogens.

## Methods

### Sex as a biological variable.

Male (*n* = 4) and female (*n* = 9) participants were recruited, and similar findings were reported for both sexes. Sex was not considered as an analysis variable.

### Participant recruitment and sampling.

Thirteen participants (31 to 76 years old) were recruited from study sites affiliated with Yale School of Medicine/Yale New Haven Hospital in New Haven, Connecticut, USA, and from Mansfield Family Practice in Storrs, Connecticut, USA. Eleven participants met the 2017 CDC criteria for LD (78) and had at least 1 EM lesion. Two additional participants with 4-cm EM lesions (one of whom had positive Lyme serologies) had probable LD based on physician-diagnosed EM in the setting of known tick bites. Participants were enrolled during the period of May to October in 2019 (*n* = 6), and in 2020 (*n* = 7). Clinical details of the participants recruited in 2019 were previously reported (see dataset 1 in ref. 18), and are included in the description of all participants in this study in Table 1.

### Sample processing.

Blood and skin samples were processed for scRNA-Seq and scTCR-Seq as previously described (18). Separate whole blood samples were collected for sera, DNA extraction, and PBMC isolation. Ficoll-Paque PLUS purified PBMCs isolated as described previously (18) were cryopreserved in 90% human AB-negative sera (GeminiBio) and 10% DMSO for bulk TCR-Seq. Three-millimeter skin punch biopsies were obtained from the leading edge of the EM lesion and from uninvolved (control) skin at least 2 cm away from the EM lesion. Biopsies were placed into MACS Tissue Storage Solution (Miltenyi Biotec, 130-100-008) for transport to the laboratory, where they were immediately processed into single-cell suspensions using the Whole Skin Dissociation Kit (Miltenyi Biotec, 130-101-540) according to the manufacturer’s recommendation, as previously described (18). Library construction from single-cell suspensions was performed using the 10x Genomics Chromium Single Cell 5′ Reagent Kit (both gene expression and immune profiling) and sequencing was done on the Illumina MiSeq platform. Sample processing produced 3 datasets: one representing samples collected in 2019 and the other 2 representing samples collected in 2020.

### scRNA-Seq data processing and quality control.

The raw sequencing data from all datasets were processed with 10x Genomics’ Cell Ranger v6.1.2 using GRCh38-2020-A as the GEX reference and vdj_GRCh38_alts_ensembl-5.0.0 as the VDJ reference (19). To have greater control over quality control decisions, *cellranger count* and *vdj* were run separately instead of *cellranger multi*. Upon checking the average read depths across the blood and skin datasets, the gene expression data were rerun using *cellranger aggr* to normalize for marked depth differences among datasets (dataset 1 had an average of 132 million reads, dataset 2 had an average of 198 million reads, and dataset 3 had an average of 390 million reads) (Supplemental Figure 13A). This subsampled reads from every GEX sample down to the lowest number of reads per cell confidently mapped to the transcriptome (Supplemental Tables 1 and 2, and Supplemental Figure 13B). An average of 5,882 cells were identified per sample across all tissues (SD = 3,742 cells), with an estimated 41,568 cells in the blood and 146,662 cells in the (combined EM and control) skin. Within the skin, there were an average of 7,221 cells (SD = 4,865 cells) and a median of 5,456 cells per participant in the EM, and an average of 4,799 cells (SD = 3,049 cells) and a median of 4,077 cells per participant in the control.

### Gene expression analysis.

The count matrices were processed using Seurat v4.4 (79) as the single-cell ecosystem. After removing low-quality cells with fewer than 200 genes and mitochondrial content greater than 15%, there were 40,733 cells remaining in the blood and 136,002 cells remaining in the skin (87,836 from the EM and 48,166 from the control). There was an average of 6,451 cells (SD = 4,359 cells) and a median of 4,972 cells per participant in the EM, and an average of 3,944 cells (SD = 2,615 cells) and a median of 3,550 cells per participant in the control skin. The data were then log-normalized with a scale.factor of 10,000 using *NormalizeData*. The top 2000 most highly variable genes were identified using the *vst* method of *FindVariableFeatures* and linearly scaled with *ScaleData*. Immunoglobulin (IG) and TCR genes were removed from the variable features using Ensembl 93 (80) as a reference for the gene names. Principal component analysis (PCA) was run on 30 PCs with *RunPCA* and then graph-based clustering was conducted on 20 dimensions (chosen based on the inflection point of an elbow plot) using *FindNeighbors* for shared nearest neighbors and *FindClusters* for Louvain-based community detection. Non-linear dimensionality reduction for visualization was done using *RunUMAP*. Default parameters were used for all functions unless otherwise specified. Both blood and skin datasets showed adequate mixings of samples, indicating no major batch effects and therefore no need to add a computational integration (harmonization) step.

Clusters were annotated using known canonical marker genes (Supplemental Figures 1 and 2). The clustering resolution was determined through iteration and refinement using the clustree package (81), with a final resolution of 1.0 being selected for the skin and 0.4 for the blood (Figures 1 and 2, and Supplemental Figure 14). We chose to initially overcluster with the understanding that similar clusters could then be combined with the same cell type label. Some clusters may contain mixtures of cell types, such as cluster 15 in the blood that we further subclustered for more accurate labeling. Cell type annotation was further supported by integrating TCR information with plots such as DotPlots to better identify T cells in both tissue types (Supplemental Figures 1 and 2, blood not shown). As 10x Genomics does not currently support the detection of γδ chains (82), we used a combination of several other approaches to define a γδ T cell cluster within the subclustered blood T cell data: reconstruction of TCRs from GEX data using TRUST4 (83), automated annotation using Azimuth (79), and expression of specific marker genes (*TRDV2*, *TRGV9*, *TRGV10*). A γδ T cell subset could not be identified within the skin.

Doublets were calculated on the level of Seurat clusters per sample and removed using scDblFinder (84). A value of 0.01 was chosen as the expected doublet rate following 10x Genomics’ documentation (1% per 1,000 captured cells). There were 38,942 cells remaining in the blood and 123,304 cells remaining in the skin (83,860 from the EM and 39,444 from the control) after doublet removal. The majority of doublets removed from the blood were from cells labeled as DCs and T cells; those from the skin were from cells labeled as fibroblasts and keratinocytes.

We identified possible Temra cells in the skin using IDEIS (identification of isoforms), a tool for assessing differentially spliced variants of CD45 in 10x Genomics data (85). This tool creates a transcriptome reference from the raw BAM files and mapping reads to the relevant exons; 17% of the skin T cells had reads successfully mapped, which is within the range reported based on reads per cell (85).

### Immune profiling analysis.

Paired-end FASTQ reads of scTCR-Seq data were processed using the *cellranger vdj* command from 10x Genomics Cell Ranger v6.1.2 (19) for alignment against the human reference in both the blood and the skin data. Filtered contig sequences were further processed using the nextflow pipeline nf-core/airrflow v4.1.0 (86), which uses tools from the Immcantation pipeline (87, 88). We used airrflow to perform V(D)J germline gene reassignment using IgBLAST v1.20.0 and the IMGT database (89, 90). Only productively rearranged sequences with valid V and J gene annotations and junction lengths divisible by 3 were retained. Cells with only α chains (no paired β chains) were discarded.

Bulk TCR-Seq data were processed as previously described (18) and combined with scTCR-Seq data to perform clonal analysis. TCR clonotypes were defined per participant as sequences sharing the same V and J genes and an identical junction region in the β chains, using the *identicalClones* function from the SCOPer package (v1.2.1) (91). Clonal overlaps were visualized using ggVennDiagram v1.5.2 (92) and clonal expansion was visualized using APackOfTheClones v1.2.4 (93).

### Subclustering.

Subclustered T cell populations were defined for the blood and the skin in several stages. In the first stage, cells already annotated as T cells on the full tissue level (Figure 1) were selected along with cells with paired TCRs, as the latter may have been real T cells that simply did not have clear enough expression to be annotated as such. After repeating the gene expression processing steps above (including removing the IG- and TCR-specific genes from the variable features again and reclustering), clusters that did not appear to be T cells based on expression of canonical T cell marker genes were removed. In the second stage, the resulting T cells were reclustered (with a resolution of 1.5 for both the blood T cells and the skin T cells) and annotated with T cell subsets using marker genes again.

For analysis of gene expression in skin, cells from the original “T Cells” group were renamed with the identified T cell subsets when possible. Any cells from the “T Cells” group that were not selected for subclustering were removed as we could not confirm that they were truly T cells.

### Differential gene expression analysis and GSEA.

To identify differentially expressed genes between control and EM samples across skin cell types, we conducted a pseudobulk analysis by aggregating cell counts for each cell type within each sample and applying DESeq2 (94). Genes were considered to be significantly differentially expressed using an FDR threshold of 0.05. Heatmaps were created using ComplexHeatmap v2.22.0 (95).

GSEA was performed on the ranked gene list from the pseudobulk analysis on the Hallmark gene sets from the Molecular Signature Database (MSigDB) (v2024.1.Hs) (96) using ClusterProfiler v4.14.4 (97). We specifically looked for gene sets that were enriched among the upregulated genes in EM (i.e., positive enrichment scores), with significance determined by a Bonferroni-corrected *P* value of less than 0.01.

### Cell-cell communication.

We inferred intercellular signaling from the scRNA-Seq data using CellChat v2.1.0 with the default CellChatDB ligand-receptor database (98, 99). A CellChat object was constructed from the count matrix (genes by cells) and cell type labels. Communication probabilities between sender and receiver groups were estimated using CellChat’s law of mass action model, which accounts for multisubunit complexes and modulators (agonists, antagonists, and coreceptors). Significant cell-cell interactions were identified with CellChat’s default parameters, using the trimean method to calculate mean gene expression. Cell types with fewer than 10 cells were excluded.

### Statistics.

All analyses were performed with R v4.4.2 (100). The significance threshold was 0.05 for *P* values (including FDR-adjusted *P* values) unless otherwise stated. A paired Wilcoxon’s signed-rank test was used to compare subset frequencies between control and EM across participants. A 2-sample *z* test for equality of proportions with continuity correction was used to compare clonal overlaps between blood and EM with blood and control skin. The control sample from participant 202943 was excluded from downstream analyses (post–T cell subset annotation) due to ambiguity in its processing.

### Study approval.

This research was conducted under human research protocol 1112009475 approved by the Yale University Institutional Review Board. Written informed consent was received from all participants prior to study inclusion.

### Data availability.

All analysis code is available at https://github.com/Kleinstein-Lab/LymeTCells Raw and processed scRNA-Seq and scTCR-Seq data used in this publication have been deposited in NCBI’s Gene Expression Omnibus (101) and are accessible at GSE297325 (https://www.ncbi.nlm.nih.gov/geo/query/acc.cgi?acc=GSE297325) and GSE169440 (https://www.ncbi.nlm.nih.gov/geo/query/acc.cgi?acc=GSE169440). The datasets are also publicly available through the ImmPort repository (https://www.immport.org) under accession number SDY3153. Values for data shown in the main and supplemental figures are reported in the Supporting Data Values file.

## Author contributions

LKB, AAB, and SHK designed the research studies. LKB, KRD, and AAB oversaw participant recruitment and sample collection. All authors participated in data acquisition, analysis, and/or interpretation, with EA, HM, and PF performing bioinformatics analysis. EA, LKB, HM, AAB, and SHK wrote the manuscript, with all authors editing and approving the final content for submission.

## Funding support

This work is the result of NIH funding, in whole or in part, and is subject to the NIH Public Access Policy. Through acceptance of this federal funding, the NIH has been given a right to make the work publicly available in PubMed Central.

- National Institute of Allergy and Infectious Diseases/NIH grants U19-AI089992 and T15LM007056.
- Harold W. Jockers Professorship (to LKB).

## Supplementary Material

Supplemental data

Supporting data values

## Figures and Tables

**Table 1:**
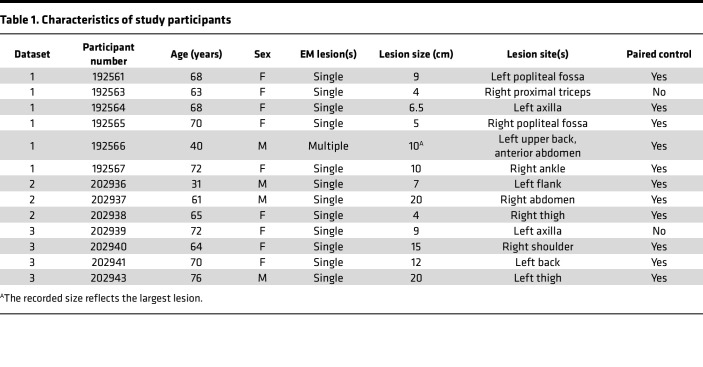
Characteristics of study participants

## Version 1 02/23/2026

Electronic publication

## References

[1] Steere AC (2016). Lyme borreliosis. Nat Rev Dis Primers.

[2] Mead P (2022). Epidemiology of Lyme disease. Infect Dis Clin North Am.

[3] Steere AC (2024). Lyme arthritis: a 50-year journey. J Infect Dis.

[4] Dirks J (2024). Disease-specific T cell receptors maintain pathogenic T helper cell responses in postinfectious Lyme arthritis. J Clin Invest.

[5] Baarsma ME, Hovius JW (2024). Persistent symptoms after Lyme disease: clinical characteristics, predictors, and classification. J Infect Dis.

[6] Bujak DI (1996). Clinical and neurocognitive features of the post Lyme syndrome. J Rheumatol.

[7] Aucott JN (2015). Posttreatment Lyme disease syndrome. Infect Dis Clin North Am.

[8] Marques A (2017). Transcriptome assessment of erythema migrans skin lesions in patients with early Lyme disease reveals predominant interferon signaling. J Infect Dis.

[9] Lochhead RB (2019). Robust interferon signature and suppressed tissue repair gene expression in synovial tissue from patients with postinfectious, Borrelia burgdorferi-induced Lyme arthritis. Cell Microbiol.

[10] Petzke MM (2020). Global transcriptome analysis identifies a diagnostic signature for early disseminated Lyme disease and its resolution. mBio.

[11] Lochhead RB (2021). Lyme arthritis: linking infection, inflammation and autoimmunity. Nat Rev Rheumatol.

[12] Glatz M (2017). Characterization of the early local immune response to Ixodes ricinus tick bites in human skin. Exp Dermatol.

[13] Müllegger RR (2000). Differential expression of cytokine mRNA in skin specimens from patients with erythema migrans or acrodermatitis chronica atrophicans. J Invest Dermatol.

[14] Salazar JC (2003). Coevolution of markers of innate and adaptive immunity in skin and peripheral blood of patients with erythema migrans. J Immunol.

[15] Böer A (2007). Erythema migrans: a reassessment of diagnostic criteria for early cutaneous manifestations of borreliosis with particular emphasis on clonality investigations. Br J Dermatol.

[17] Debes GF, McGettigan SE (2019). Skin-associated B cells in health and inflammation. J Immunol.

[18] Jiang R (2021). Single cell immunophenotyping of the skin lesion erythema migrans identifies IgM memory B cells. JCI Insight.

[19] Zheng GXY (2017). Massively parallel digital transcriptional profiling of single cells. Nat Commun.

[20] Vorstandlechner V (2020). Deciphering the functional heterogeneity of skin fibroblasts using single-cell RNA sequencing. FASEB J.

[21] Alagar Boopathy LR (2022). Mechanisms tailoring the expression of heat shock proteins to proteostasis challenges. J Biol Chem.

[22] Shen S (2010). Treg cell numbers and function in patients with antibiotic-refractory or antibiotic-responsive Lyme arthritis. Arthritis Rheum.

[23] Hovhannisyan Z (2011). Characterization of interleukin-17-producing regulatory T cells in inflamed intestinal mucosa from patients with inflammatory bowel diseases. Gastroenterology.

[24] Feng T (2011). Interleukin-12 converts Foxp3^+^ regulatory T cells to interferon-γ-producing Foxp3^+^ T cells that inhibit colitis. Gastroenterology.

[25] Koenecke C (2012). IFN-γ production by allogeneic Foxp3^+^ regulatory T cells is essential for preventing experimental graft-versus-host disease. J Immunol.

[26] Roncarolo MG (2018). The biology of T regulatory type 1 cells and their therapeutic application in immune-mediated diseases. Immunity.

[27] Fujioka S (2025). Single-cell multiomic analysis revealed the differentiation, localization, and heterogeneity of IL10+ Foxp3– follicular T cells in humans. Int Immunol.

[28] Song Y (2021). Tr1 cells as a key regulator for maintaining immune homeostasis in transplantation. Front Immunol.

[29] Edwards CL (2023). IL-10-producing Th1 cells possess a distinct molecular signature in malaria. J Clin Invest.

[30] Chen PP (2021). Alloantigen-specific type 1 regulatory T cells suppress through CTLA-4 and PD-1 pathways and persist long-term in patients. Sci Transl Med.

[31] Verhagen J (2006). Absence of T-regulatory cell expression and function in atopic dermatitis skin. J Allergy Clin Immunol.

[32] Müllegger RR (2007). Chemokine signatures in the skin disorders of Lyme borreliosis in Europe: predominance of CXCL9 and CXCL10 in erythema migrans and acrodermatitis and CXCL13 in lymphocytoma. Infect Immun.

[33] Jonsson AH (2022). Granzyme K^+^ CD8 T cells form a core population in inflamed human tissue. Sci Transl Med.

[34] Knörck A (2022). Cytotoxic efficiency of human CD8^+^ T cell memory subtypes. Front Immunol.

[35] Krummey SM (2020). CD45RB status of CD8^+^ T cell memory defines T cell receptor affinity and persistence. Cell Rep.

[36] Mosser DD (1997). Role of the human heat shock protein hsp70 in protection against stress-induced apoptosis. Mol Cell Biol.

[37] Chand K (2021). Comparative analysis of differential gene expression of HSP40 and HSP70 family isoforms during heat stress and HIV-1 infection in T-cells. Cell Stress Chaperones.

[38] Figueiredo C (2009). Heat shock protein 70 (HSP70) induces cytotoxicity of T-helper cells. Blood.

[39] Hirano N (2006). Engagement of CD83 ligand induces prolonged expansion of CD8^+^ T cells and preferential enrichment for antigen specificity. Blood.

[40] Jin F (2019). The orphan nuclear receptor NR4A1 promotes FcεRI-stimulated mast cell activation and anaphylaxis by counteracting the inhibitory LKB1/AMPK axis. Allergy.

[41] Shin HJ (2009). T-bet expression is regulated by EGR1-mediated signaling in activated T cells. Clin Immunol.

[42] Suurväli J (2015). RGS16 restricts the pro-inflammatory response of monocytes. Scand J Immunol.

[43] Schmetterer KG (2019). Overexpression of PDE4A acts as checkpoint inhibitor against cAMP-mediated immunosuppression in vitro. Front Immunol.

[44] Filgueira L (1996). Human dendritic cells phagocytose and process Borrelia burgdorferi. J Immunol.

[45] Shi Z (2024). The role of dermal fibroblasts in autoimmune skin diseases. Front Immunol.

[46] Cavagnero KJ, Gallo RL (2022). Essential immune functions of fibroblasts in innate host defense. Front Immunol.

[47] Kersh AE (2024). CXCL9, CXCL10, and CCL19 synergistically recruit T lymphocytes to skin in lichen planus. JCI Insight.

[48] Duray PH (1989). Histopathology of clinical phases of human Lyme disease. Rheum Dis Clin North Am.

[49] Crawford A (2011). A role for the chemokine RANTES in regulating CD8 T cell responses during chronic viral infection. PLoS Pathog.

[50] Syed M (2025). The multifaceted role of XCL1 in health and disease. Protein Sci.

[51] Bhat MY (2018). Comprehensive network map of interferon gamma signaling. J Cell Commun Signal.

[52] Mathy NL (2000). Interleukin-16 stimulates the expression and production of pro-inflammatory cytokines by human monocytes. Immunology.

[53] Lee J (2022). Tissue resident Foxp3^+^ regulatory T cells: sentinels and saboteurs in health and disease. Front Immunol.

[54] Aass KR (2021). Molecular interactions and functions of IL-32. J Leukoc Biol.

[55] Derlindati E (2015). Transcriptomic analysis of human polarized macrophages: more than one role of alternative activation?. PLoS One.

[56] Kitsou C (2021). Tick host immunity: vector immunomodulation and acquired tick resistance. Trends Immunol.

[57] Kleissl L (2025). Ticks’ tricks: immunomodulatory effects of ixodid tick saliva at the cutaneous tick-host interface. Front Immunol.

[58] Poon MML (2023). Tissue adaptation and clonal segregation of human memory T cells in barrier sites. Nat Immunol.

[59] Hamann D (1997). Phenotypic and functional separation of memory and effector human CD8^+^ T cells. J Exp Med.

[60] Zhang F (2019). Defining inflammatory cell states in rheumatoid arthritis joint synovial tissues by integrating single-cell transcriptomics and mass cytometry. Nat Immunol.

[61] Duquette D (2023). Human granzyme K is a feature of innate T cells in blood, tissues, and tumors, responding to cytokines rather than TCR stimulation. J Immunol.

[62] Donado CA (2025). Granzyme K activates the entire complement cascade. Nature.

[63] Sol S (2024). Unraveling the functional heterogeneity of human skin at single-cell resolution. Hematol Oncol Clin North Am.

[64] Meddeb M (2016). Homogeneous inflammatory gene profiles induced in human dermal fibroblasts in response to the three main species of Borrelia burgdorferi sensu lato. PLoS One.

[65] Ball HJ (2014). Tryptophan-catabolizing enzymes – party of three. Front Immunol.

[66] Opitz CA (2011). An endogenous tumour-promoting ligand of the human aryl hydrocarbon receptor. Nature.

[67] Shinde R (2015). B cell-intrinsic IDO1 regulates humoral immunity to T cell-independent antigens. J Immunol.

[68] Muehlenbachs A (2016). Cardiac tropism of Borrelia burgdorferi: an autopsy study of sudden cardiac death associated with Lyme carditis. Am J Pathol.

[69] Bertrand E (1999). Central nervous system infection caused by Borrelia burgdorferi. Clinico-pathological correlation of three post-mortem cases. Folia Neuropathol.

[70] Jacobsen M (2003). Clonal accumulation of activated CD8+ T cells in the central nervous system during the early phase of neuroborreliosis. J Infect Dis.

[71] Brissette CA (2013). The multifaceted responses of primary human astrocytes and brain microvascular endothelial cells to the Lyme disease spirochete, Borrelia burgdorferi. ASN Neuro.

[72] Lochhead RB (2019). Interferon-gamma production in Lyme arthritis synovial tissue promotes differentiation of fibroblast-like synoviocytes into immune effector cells. Cell Microbiol.

[73] Rouse JR (2024). HLA-DR-expressing fibroblast-like synoviocytes are inducible antigen presenting cells that present autoantigens in Lyme arthritis. ACR Open Rheumatol.

[74] Strobl J (2022). Tick feeding modulates the human skin immune landscape to facilitate tick-borne pathogen transmission. J Clin Invest.

[75] Nordberg M (2011). Cytotoxic mechanisms may play a role in the local immune response in the central nervous system in neuroborreliosis. J Neuroimmunol.

[76] Busch DH (1996). Detection of Borrelia burgdorferi-specific CD8^+^ cytotoxic T cells in patients with Lyme arthritis. J Immunol.

[77] Ordóñez D (2023). Cell-mediated cytotoxicity in Lyme arthritis. Arthritis Rheumatol.

[78] https://ndc.services.cdc.gov/case-definitions/lyme-disease-2017/.

[79] Hao Y (2021). Integrated analysis of multimodal single-cell data. Cell.

[80] Dyer SC (2025). Ensembl 2025. Nucleic Acids Res.

[81] Zappia L, Oshlack A (2018). Clustering trees: a visualization for evaluating clusterings at multiple resolutions. Gigascience.

[82] https://kb.10xgenomics.com/hc/en-us/articles/360015793931-Can-I-detect-T-cells-with-gamma-delta-chains-in-my-V-D-J-data.

[83] Song L (2021). TRUST4: immune repertoire reconstruction from bulk and single-cell RNA-seq data. Nat Methods.

[84] Germain P (2021). Doublet identification in single-cell sequencing data using scDblFinder. F1000Res.

[85] Michalik J (2024). IDEIS: a tool to identify PTPRC/CD45 isoforms from single-cell transcriptomic data. Front Immunol.

[86] Gabernet G (2024). nf-core/airrflow: An adaptive immune receptor repertoire analysis workflow employing the Immcantation framework. PLoS Comput Biol.

[87] Gupta NT (2015). Change-O: a toolkit for analyzing large-scale B cell immunoglobulin repertoire sequencing data. Bioinformatics.

[88] Vander Heiden JA (2014). pRESTO: a toolkit for processing high-throughput sequencing raw reads of lymphocyte receptor repertoires. Bioinformatics.

[89] Ye J (2013). IgBLAST: an immunoglobulin variable domain sequence analysis tool. Nucleic Acids Res.

[90] Giudicelli V (2005). IMGT/GENE-DB: a comprehensive database for human and mouse immunoglobulin and T cell receptor genes. Nucleic Acids Res.

[91] Nouri N, Kleinstein SH (2018). A spectral clustering-based method for identifying clones from high-throughput B cell repertoire sequencing data. Bioinformatics.

[92] Gao CH (2024). ggVennDiagram: intuitive Venn diagram software extended. Imeta.

[93] Yang Q (2024). PulPy: A Python Dolkit for MRI RF aDign. J Open Source Softw.

[94] Squair JW (2021). Confronting false discoveries in single-cell differential expression. Nat Commun.

[95] Gu Z (2022). Complex heatmap visualization. Imeta.

[96] Liberzon A (2015). The Molecular Signatures Database (MSigDB) hallmark gene set collection. Cell Syst.

[97] Yu G (2012). clusterProfiler: an R package for comparing biological themes among gene clusters. OMICS.

[98] Jin S (2025). CellChat for systematic analysis of cell-cell communication from single-cell transcriptomics. Nat Protoc.

[99] Jin S (2021). Inference and analysis of cell-cell communication using CellChat. Nat Commun.

[100] https://www.R-project.org/.

[101] Edgar R (2002). Gene Expression Omnibus: NCBI gene expression and hybridization array data repository. Nucleic Acids Res.

