# Supplementary material for: Single-cell immunophenotyping identifies CD8+GZMK+IFNG+ T cells as a key immune population in cutaneous Lyme disease
Source: JCI Insight. 2026 Feb 23;11(4):e196741. doi: 10.1172/jci.insight.196741 (PMC12956012; doi:10.1172/jci.insight.196741)
Supplement: Supplemental data [file jciinsight-11-196741-s228.pdf]

Supplemental Table 1. Sequencing Metrics for the EM and Control Skin Samples

Sample	Participant Number	Sample Type	Read Count	Cell Count	Reads per Cell	Median Genes per Cell	Cell Count (TCR)
192561SKL	192561	EM	73,517,541	4,007	30,263	1,173	1,235
192561SKN	192561	Control	71,570,670	4,077	27,799	1,457	123
192563SKL	192563	EM	67,594,466	694	177,215	1,387	97
192564SKL	192564	EM	68,158,835	11,910	11,132	775	2,461
192564SKN	192564	Control	47,310,535	332	395,201	1,406	2
192565SKL	192565	EM	66,997,095	14,068	9,426	617	4,562
192565SKN	192565	Control	47,234,285	1,406	80,816	1,472	107
192566SKL	192566	EM	65,338,261	11,029	14,839	1,076	1,839
192566SKN	192566	Control	112,369,682	11,026	11,293	512	182
192567SKL	192567	EM	76,029,097	14,976	8,241	638	2,954
192567SKN	192567	Control	76,003,998	7,913	16,585	1,110	1,049
202936_SC	202936	Control	108,777,626	4,072	41,666	1,326	226
202936_EM	202936	EM	89,915,321	4,531	43,996	1,157	477
202937_SC	202937	Control	101,531,921	5,093	46,306	1,609	171
202937_EM	202937	EM	139,186,282	4,551	37,739	1,510	523
202938_EM	202938	EM	130,104,124	4,577	45,263	1,494	1,712
202938_SC	202938	Control	134,091,428	2,706	73,706	2,249	7
202939_EM	202939	EM	300,648,107	5,456	78,205	3,424	165
202940_EM	202940	EM	265,661,027	5,646	72,362	1,680	1,621
202940_SC	202940	Control	230,981,933	3,963	85,814	2,126	444
202941_EM	202941	EM	166,496,693	798	467,776	560	51
202941_SC	202941	Control	237,068,215	4,797	79,385	1,645	201
202943_EM	202943	EM	229,541,889	11,627	36,143	793	317
202943_SC	202943	Control	229,099,404	7,407	54,020	1,340	1,079

Values reported for the read count, reads per cell and median genes per cell are for the data prior to aggregation.

Supplemental Table 2. Sequencing Metrics for the Blood Samples

Sample	Participant Number	Sample Type	Read Count	Cell Count	Reads per Cell	Median Genes per Cell	Cell Count (TCR)
192566	192566	Blood	101,373,140	5,500	31,600	1,382	3,296
202936_B	202936	Blood	116,974,776	3,967	49,373	1,163	1,198
202937_B	202937	Blood	126,658,816	4,710	40,983	1,212	1,841
202938_B	202938	Blood	134,372,337	4,781	44,552	1,067	2,103
202939_B	202939	Blood	270,784,961	3,691	114,497	1,304	1,318
202940_B	202940	Blood	231,670,960	4,372	80,181	1,296	2,022
202941_B	202941	Blood	264,634,302	5,960	66,175	1,262	2,649
202943_B	202943	Blood	238,035,535	8,587	42,965	1,042	3,478

Values reported for the read count, reads per cell and median genes per cell are for the data prior to aggregation.

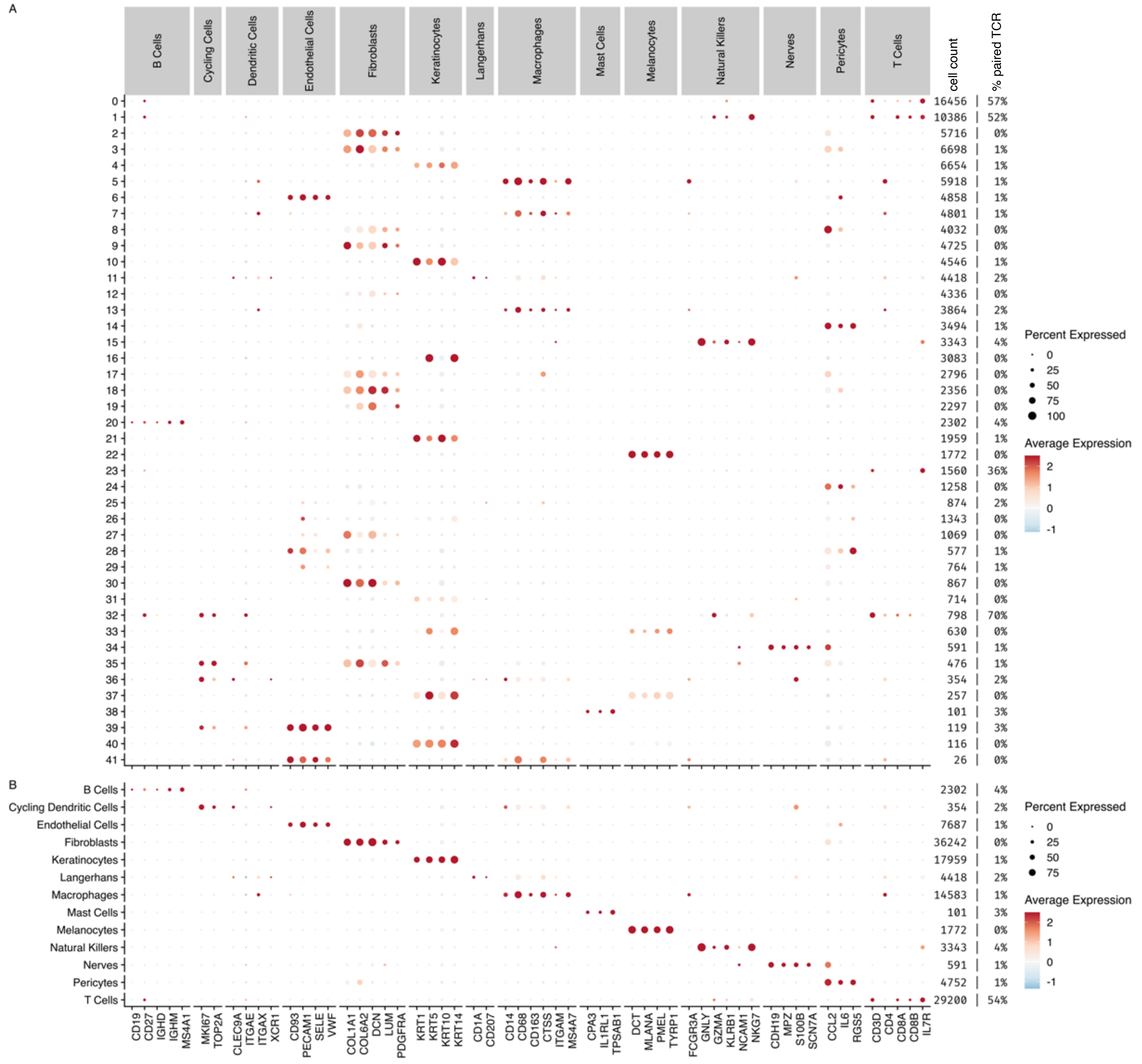

Supplemental Figure 1. Clustering and annotation of the skin. A) Dot plot showing the average expression of characteristic marker genes for each cluster within the skin. **B)** Dot plot showing the average expression of the same set of markers for each annotated cell type within the skin. Clusters were annotated based upon marker gene expression within each cluster: B cells (cluster 20), cycling dendritic cells (cluster 36), endothelial cells (clusters 6, 26, 28, 29, 39, and 41), fibroblasts (clusters 2, 3, 8, 9, 12, 17, 18, 19, 25, 27, 30, and 35), keratinocytes (clusters 4, 10, 16, 21, 31, 33, 37, and 40), Langerhans (cluster 11), macrophages (clusters 5, 7, and 13), mast cells (cluster 38), melanocytes (cluster 22), NK cells (cluster 15), nerves (cluster 34), pericytes (clusters 14 and 24), and T cells (clusters 0, 1, 23, and 32). Red dots indicate higher levels of expression and blue dots indicate lower levels of expression, with the size of the dot reflecting the percentage of that cluster of cell type that expressed the corresponding marker. Numbers in the two columns on the right side of the plots indicate the total number of cells in that cluster or cell type and the percentage that have a paired TCR, respectively. The gray rectangles on top of the dot plots are visual aids for grouping marker genes.

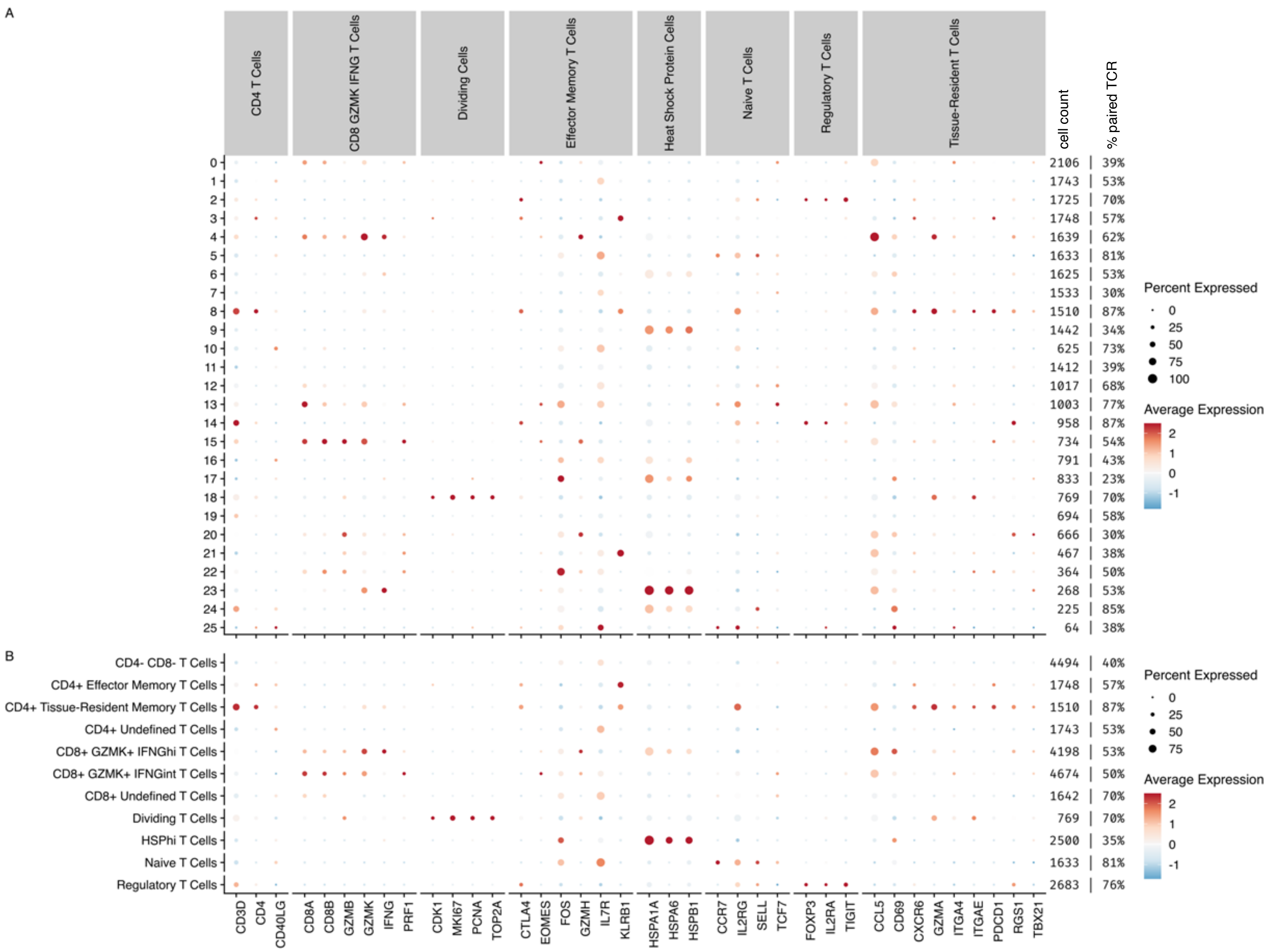

Supplemental Figure 2. Clustering and annotation of the skin T cells. A) Dot plot showing the average expression of characteristic marker genes for each cluster within the skin T cells. **B)** Dot plot showing the average expression of the same set of markers for each annotated cell type within the skin. Clusters were annotated based upon marker gene expression within each cluster: CD4$^-$ CD8$^-$ T cells (clusters 7, 11, 16, 19, and 25), CD4$^+$ effector memory T cells (cluster 3), CD4$^+$ tissue-resident memory T cells (cluster 8), CD4$^+$ undefined T cells (cluster 1), CD8$^+$GZMK$^+$IFNG^{hi} T cells (clusters 4, 6, 20, and 23), CD8$^+$GZMK$^+$IFNG^{int} T cells (clusters 0, 13, 15, 21, and 22), CD8$^+$ undefined T cells (clusters 10 and 12), dividing T cells (cluster 18), HSP^{hi} T cells (clusters 9, 17, and 24), naive T cells (cluster 5), and Tregs (clusters 2 and 14). Red dots indicate higher levels of expression and blue dots indicate lower levels of expression, with the size of the dot reflecting the percentage of that cluster of cell type that expressed the corresponding marker. The two columns of information on the right side of the plots indicate the total number of cells in that cluster or cell type and the percentage that have a paired TCR, respectively. The gray rectangles on top of the dot plots are visual aids for grouping marker genes.

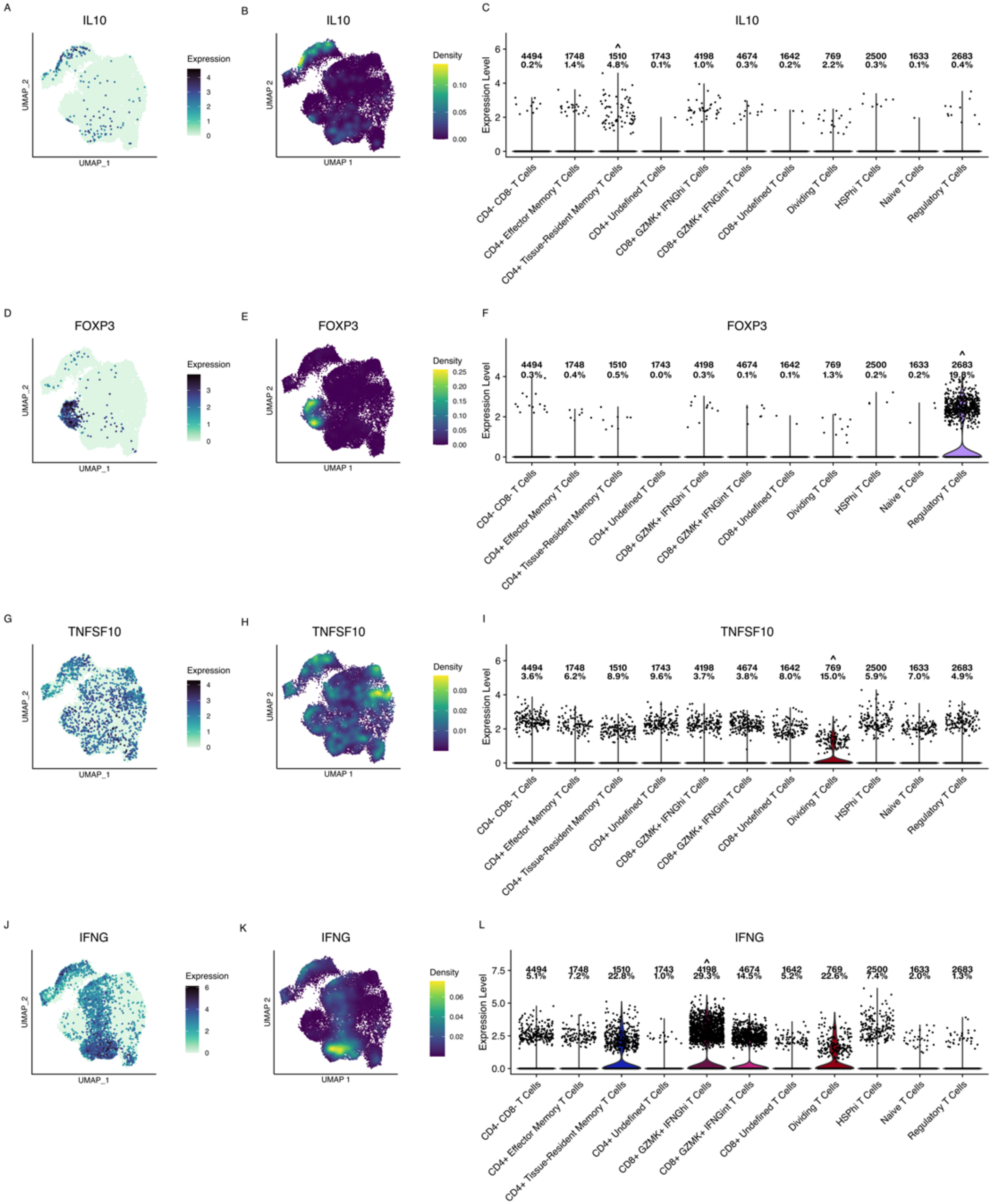

Supplemental Figure 3. Expression of select genes within the skin T cells. Each row shows the expression of *IL10* **(A-C)**, *FOXP3* **(D-F)**, *TNFSF10* **(G-I)**, and *IFNG* **(J-L)**, respectively. A, D, G, and J show the average expression of each gene upon the skin T cells UMAP, with darker colors corresponding to higher expression. B, E, H, and L show expression as a feature of kernel density using the Nebulosa package to allow for better visualization. C, F, H, and L show expression on a cell type level, with points representing individual cells. The top row of numbers within the violin plots is a count of how many cells are in that cell type, and the second row is the percentage of them with positive expression of the relative gene.

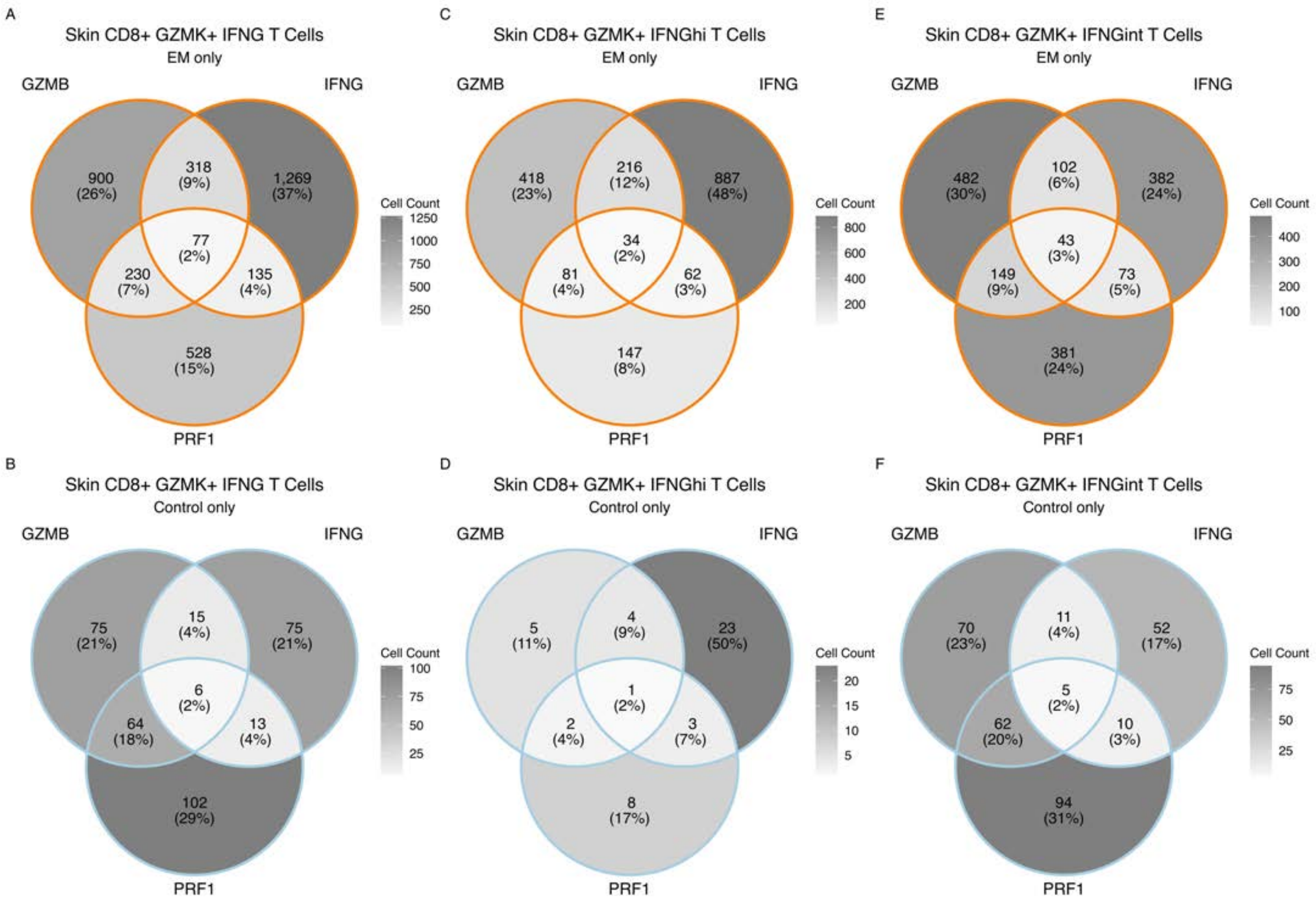

Supplemental Figure 4. Positive expression overlaps of *GZMB*, *IFNG*, and *PRF1* within the CD8$^+$ GZMK$^+$IFNG$^+$ T cells. Each Venn diagram represents the number of cells with positive expression of *GZMB*, *IFNG*, and/or *PRF1*, with percentages relative to the total cells being represented in that Venn diagram. The top row is for only the EM subset, and the bottom row is for only the control subset. **A)** and **B)** are for both cell types combined, **C)** and **D)** are for the CD8$^+$GZMK$^+$IFNG^{hi} T cells, and **E)** and **F)** are for the CD8$^+$GZMK$^+$IFNG^{int} T cells.

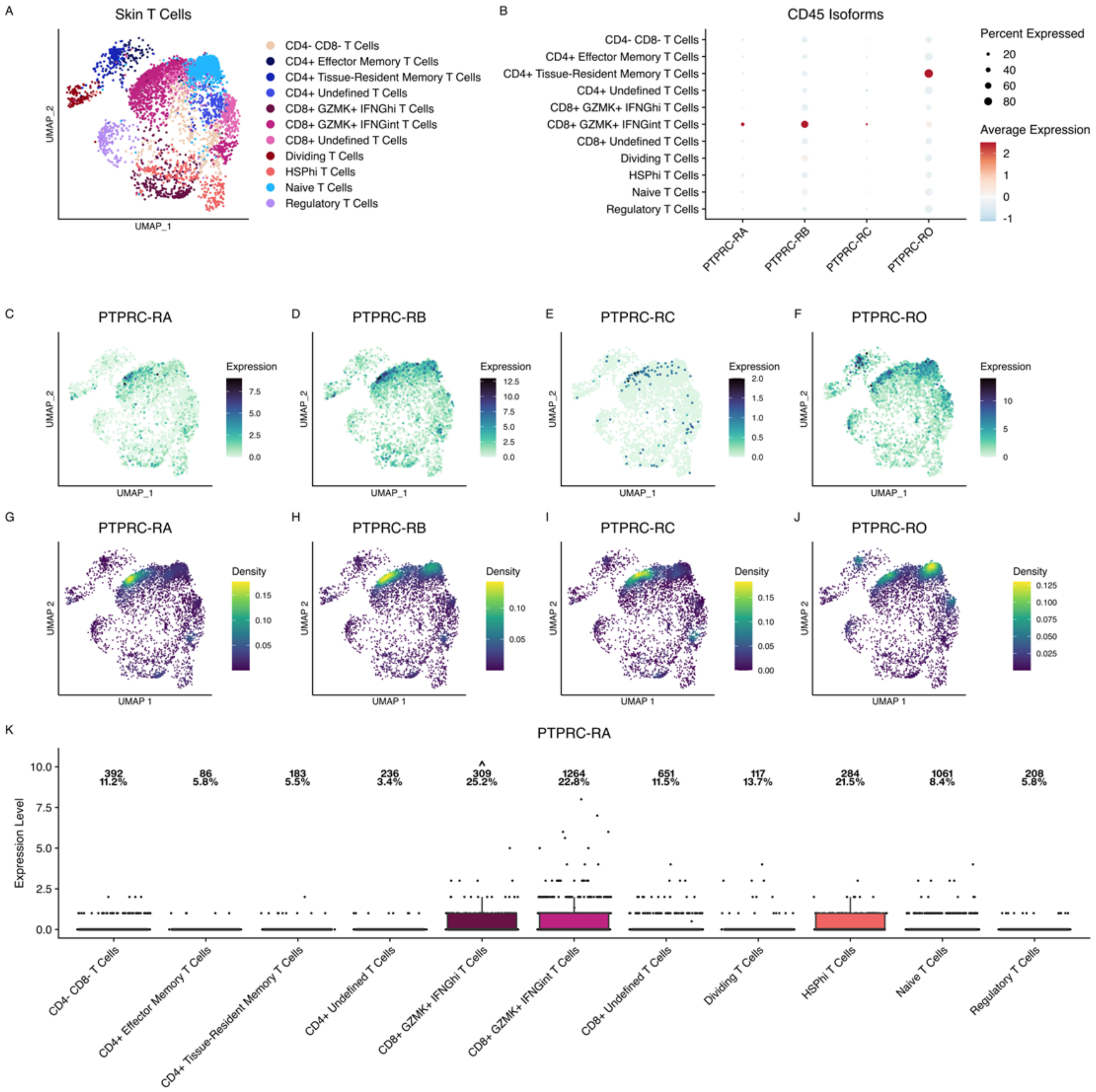

Supplemental Figure 5. *CD45* spliced isoforms in skin T cells using IDEIS. A) UMAP showing skin T cells that had a spliced isoform of *PTPRC/CD45* (RA, RB, RC, or RO) detected with the IDEIS package. **B)** Dot plot showing the expression of each isoform per T cell subtype. **C-F)** Expression of each isoform upon the skin T cells UMAP, with darker colors corresponding to higher expression. **G-L)** Expression as a feature of kernel density using the Nebulosa package to allow for better visualization. **K)** Expression on a cell type level, with points representing individual cells. The top row of numbers within the box plot is a count of how many cells are in that cell type, and the second row is the percentage of them with positive expression of *CD45RA*.

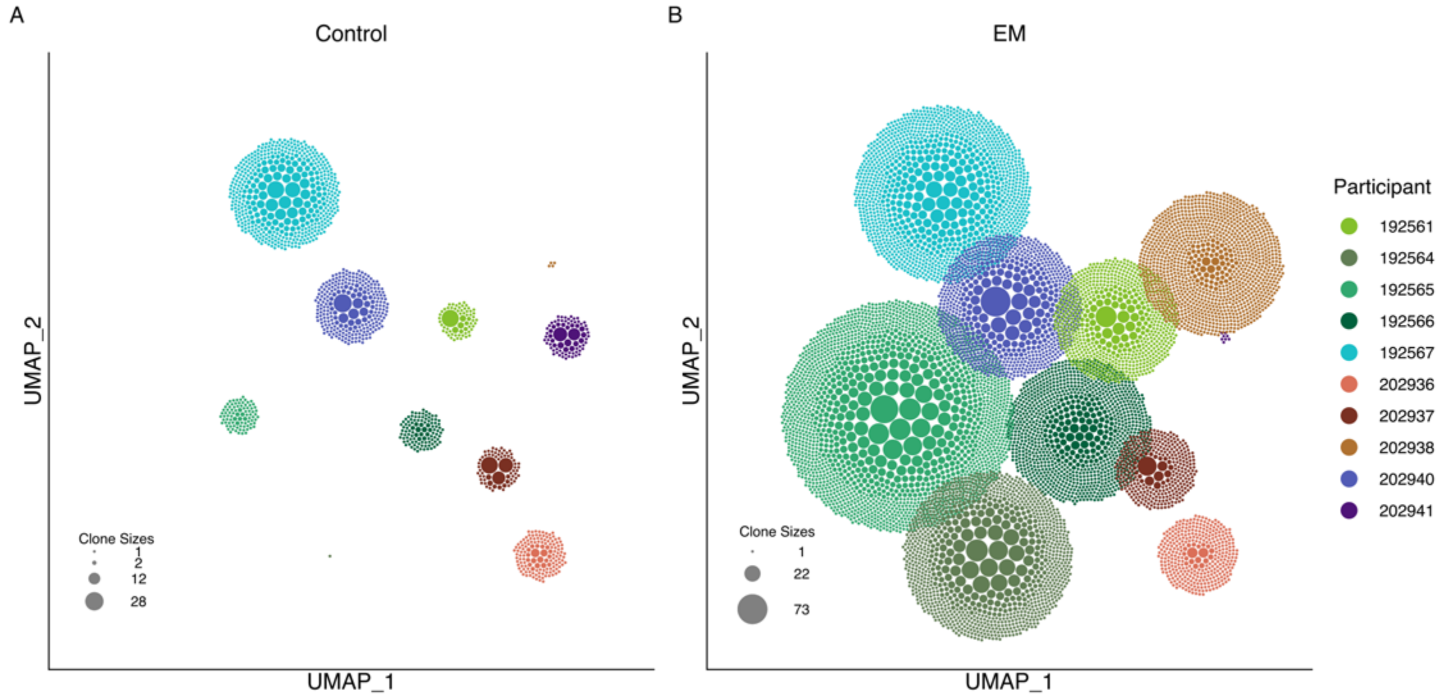

Supplemental Figure 6. Clonal expansion of skin T cells. A) and **B)** UMAP of the skin T cells in the control and EM skin, respectively, with points representing clonotype sizes within each participant. The scale of the point size is consistent for both sample types, as is the location of the centroid of each group. Participants without paired control and EM samples are not shown.

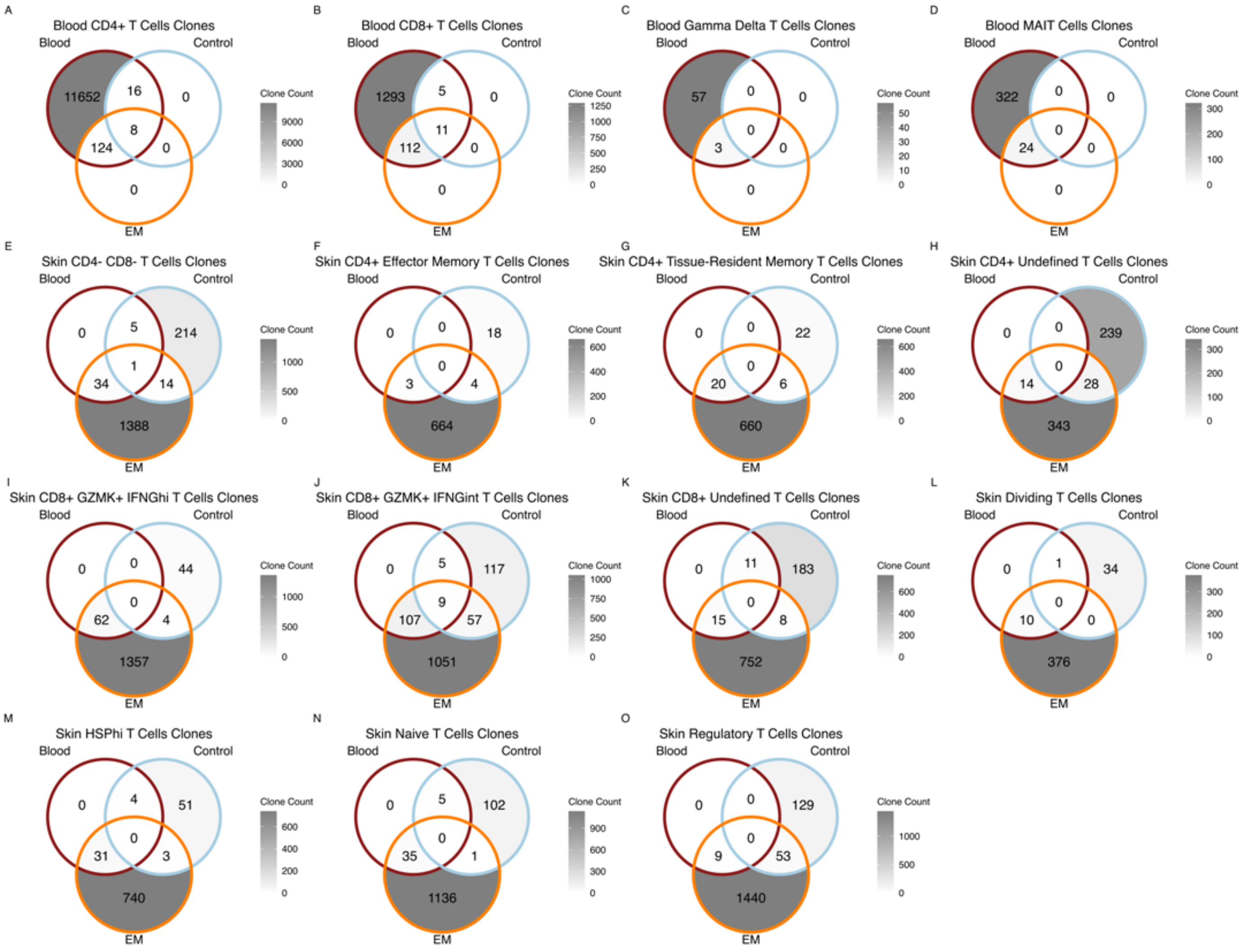

Supplemental Figure 7. T cell clonal overlaps between the blood and the skin. A-D) Clonal overlaps between 4 major blood T cell subtypes ($CD4^+$, $CD8^+$, γδ T cells, and MAIT cells) and clonotypes within the EM and control skin samples. **E-O)** Clonotypes identified within each of the 11 defined T cell subtypes in EM and control skin samples that shared TCRs with blood samples. Values represent the number of unique TCRs identified per tissue. Counts for the clonotypes only in skin T cells (A-D) and blood T cells (E-O) are zero because as only clonotypes for specified blood or skin cell types respectively are counted.

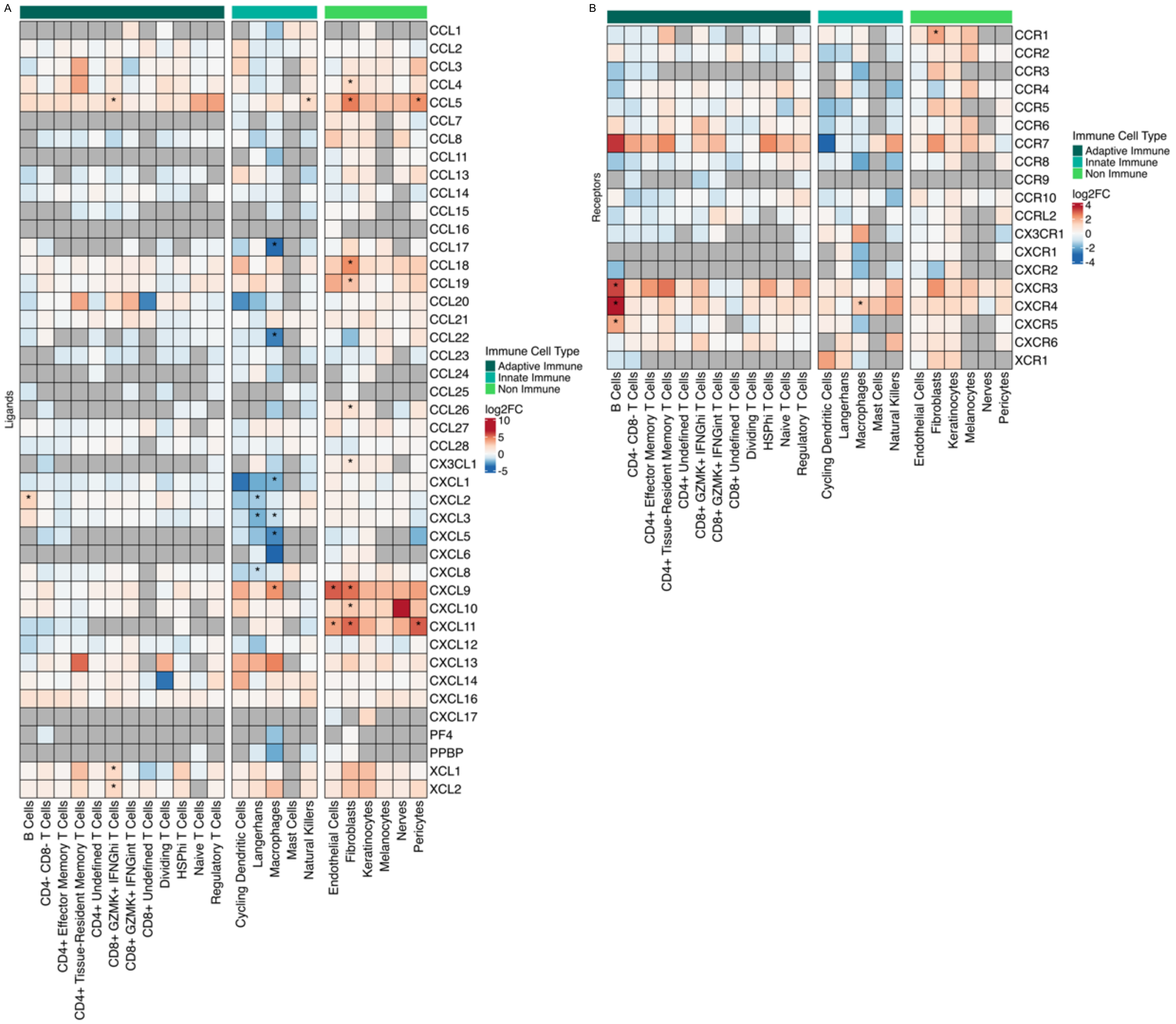

Supplemental Figure 8. Expression of chemokine ligand and receptor genes in the skin. Heatmap showing the average fold change of the expression of all chemokine ligand **(A)** and receptor **(B)** genes across the full set of skin cell types. The bar on the top designates cell types as immune- or non-immune related (dark green for adaptive immune cell types, cyan for innate immune cell types, and bright green for non-immune cell types). Within the heatmap, red indicates upregulation, and blue indicates downregulation, with asterisks denoting statistically significant changes (FDR < 0.05). Gray cells, if present, indicate that gene was filtered out by DESeq2 for that cell type. Differential expression was calculated between the EM and control portions of each cell type. Note that *PF4* is *CXCL4* and *PPBP* is *CXCL7*.

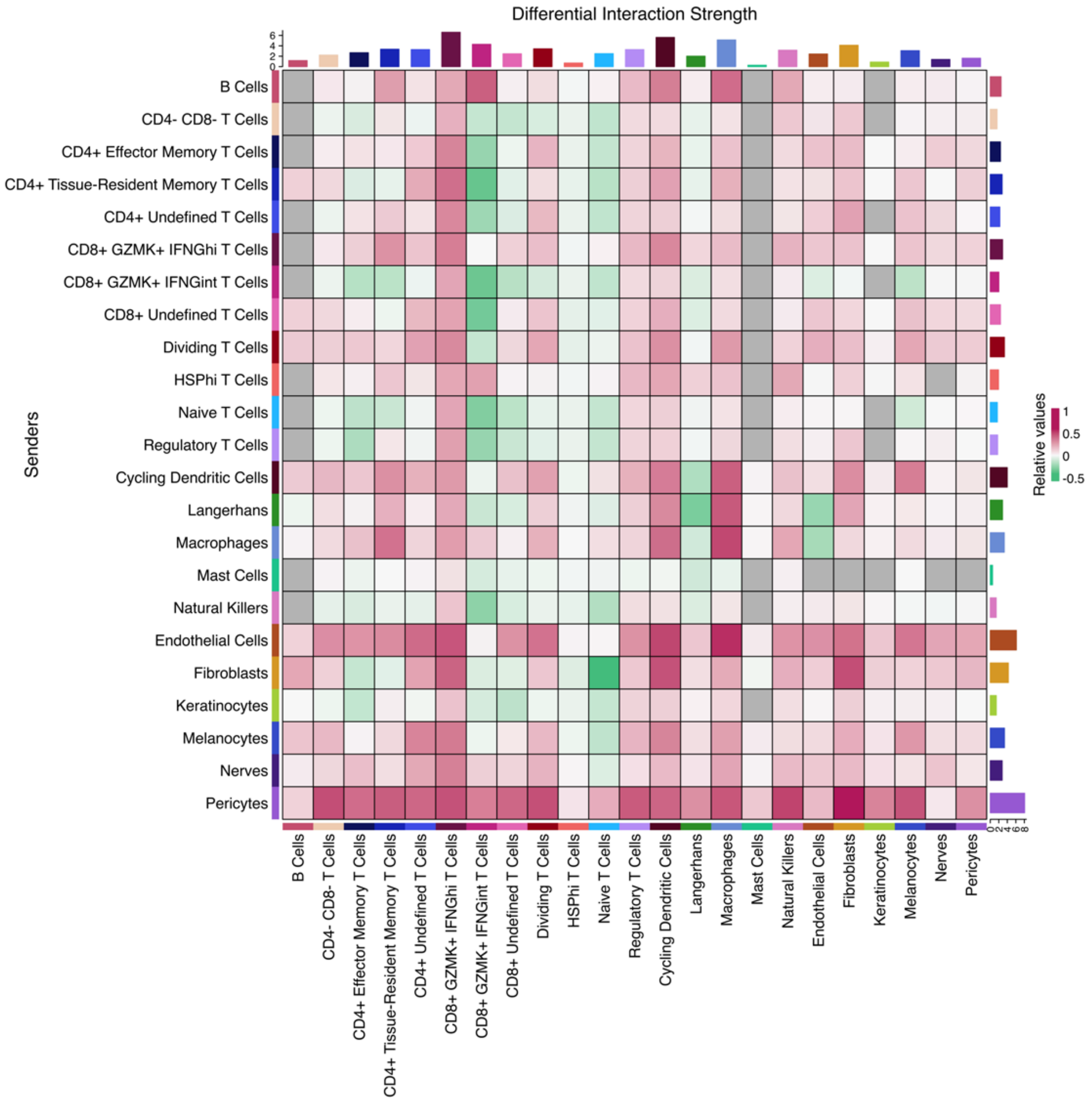

Supplemental Figure 9. Strength of differential interactions among skin cell types. Heatmap showing the increased (pink) or decreased (green) interaction strength between the sender cell types (y-axis) and the receiver cell types (x-axis). The bar plots on the top and the right sides represent the sum of the absolute values of the strength in the corresponding columns or rows respectively. The colors on all sides correspond to the cell type colors in Figures 1 and 2. Cell types are in the same order as the other heatmaps.

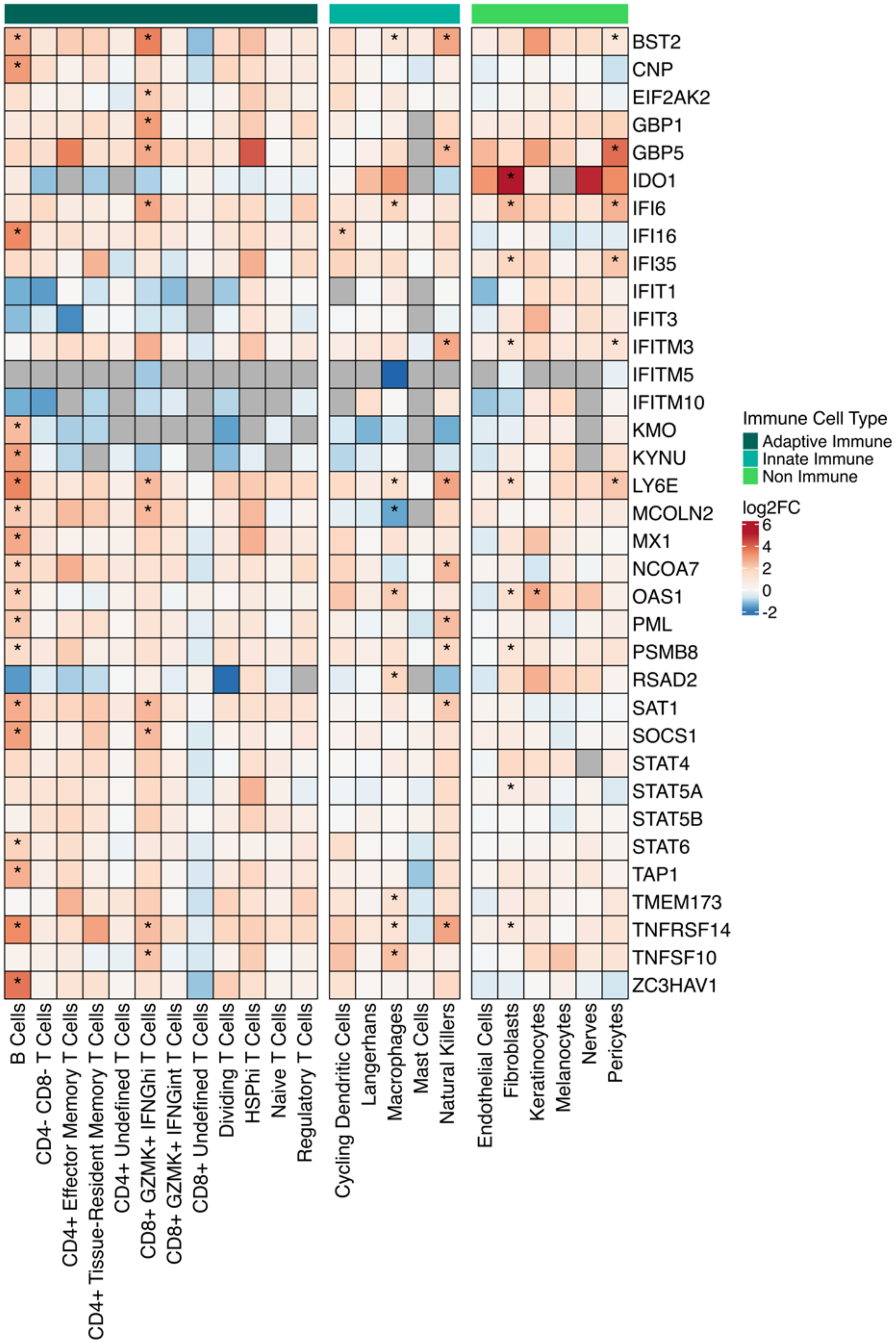

Supplemental Figure 10. Expression of select interferon-regulated genes in the skin. Heatmap showing the average fold change of the expression of relevant genes across the full set of skin cell types. The bar on the top designates cell types as immune- or nonimmune related (dark green for adaptive immune cell types, cyan for innate immune cell types, and bright green for nonimmune cell types). Within the heatmap, red indicates upregulation, and blue indicates downregulation, with asterisks denoting statistically significant changes (FDR < 0.05). Gray cells, if present, indicate that gene was filtered out by DESeq2 for that cell type. Differential expression was calculated between the EM and control portions of each cell type.

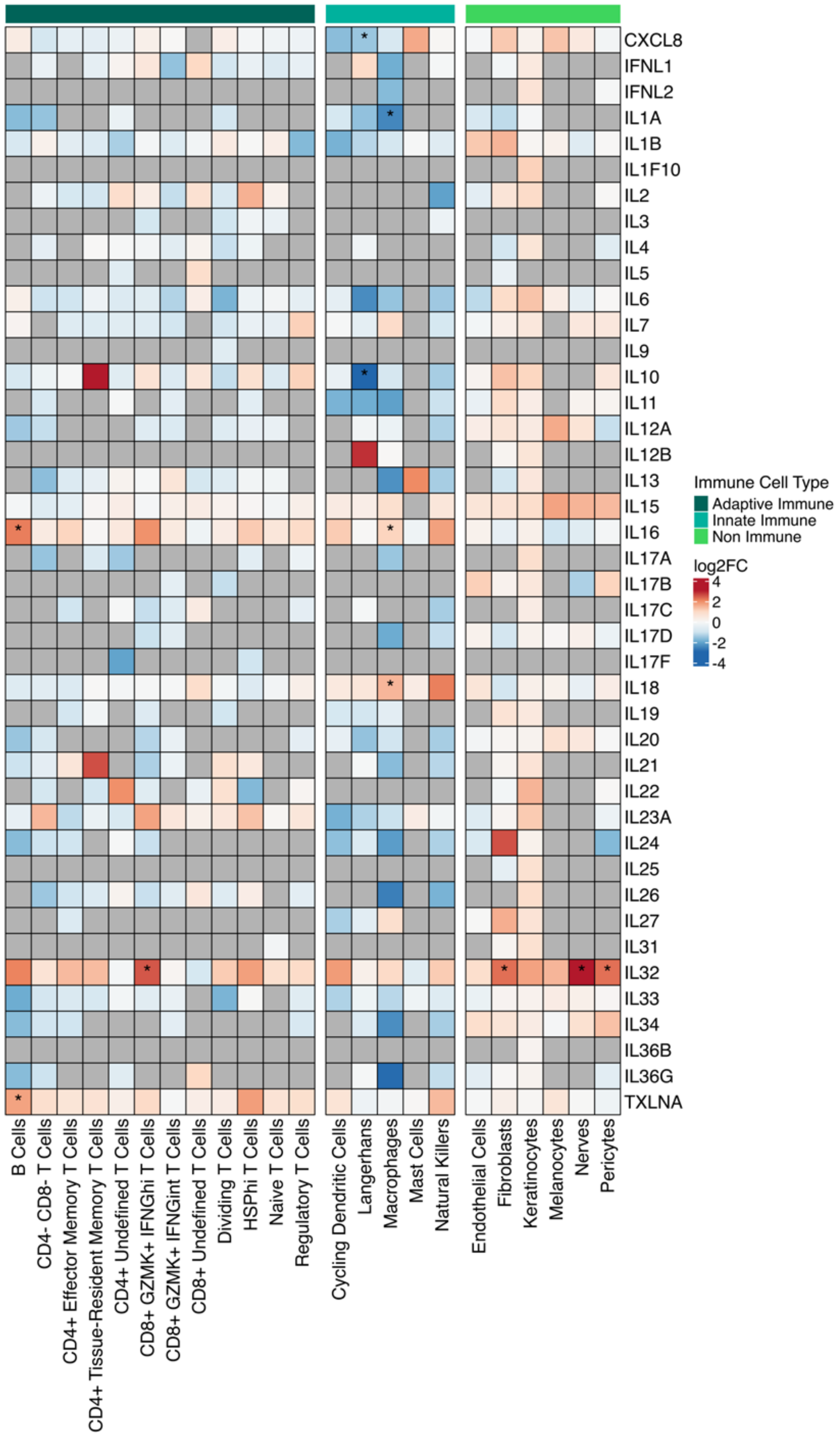

Supplemental Figure 11. Expression of interleukin genes in the skin. Heatmap showing the average fold change of the expression of all interleukin genes (except for receptor agonists) across the full set of skin cell types. The bar on the top designates cell types as immune- or nonimmune related (dark green for adaptive immune cell types, cyan for innate immune cell types, and bright green for nonimmune cell types). Within the heatmap, red indicates upregulation, and blue indicates downregulation, with asterisks denoting statistically significant changes (FDR < 0.05). Gray cells, if present, indicate that gene was filtered out by DESeq2 for that cell type. Differential expression was calculated between the EM and control portions of each cell type. Note that *CXCL8* is *IL8*, *TXLNA* is *IL14*, *IFNL1* is *IL29*, and *IFNL2* is *IL28A*.

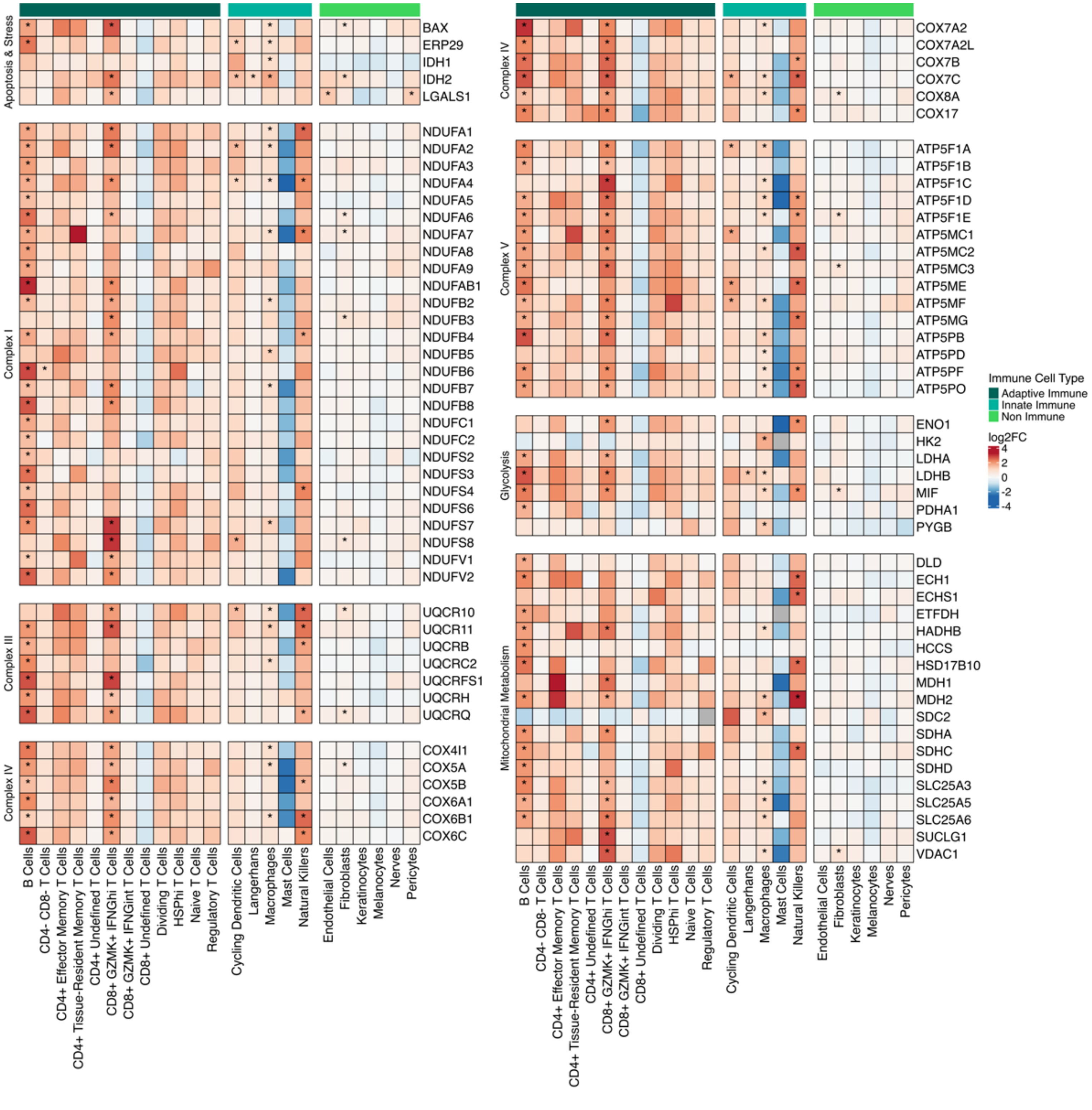

Supplemental Figure 12. Expression of metabolism-related genes in the skin. Heatmap showing the average fold change of the expression of genes significant in at least one cell type from MSigDB's Hallmark glycolysis and oxidative phosphorylation pathways across the full set of skin cell types. The bar on the top designates cell types as immune- or nonimmune related (dark green for adaptive immune cell types, cyan for innate immune cell types, and bright green for nonimmune cell types). Within the heatmap, red indicates upregulation, and blue indicates downregulation, with asterisks denoting statistically significant changes (FDR < 0.05). Gray cells, if present, indicate that gene was filtered out by DESeq2 for that cell type. Differential expression was calculated between the EM and control portions of each cell type.

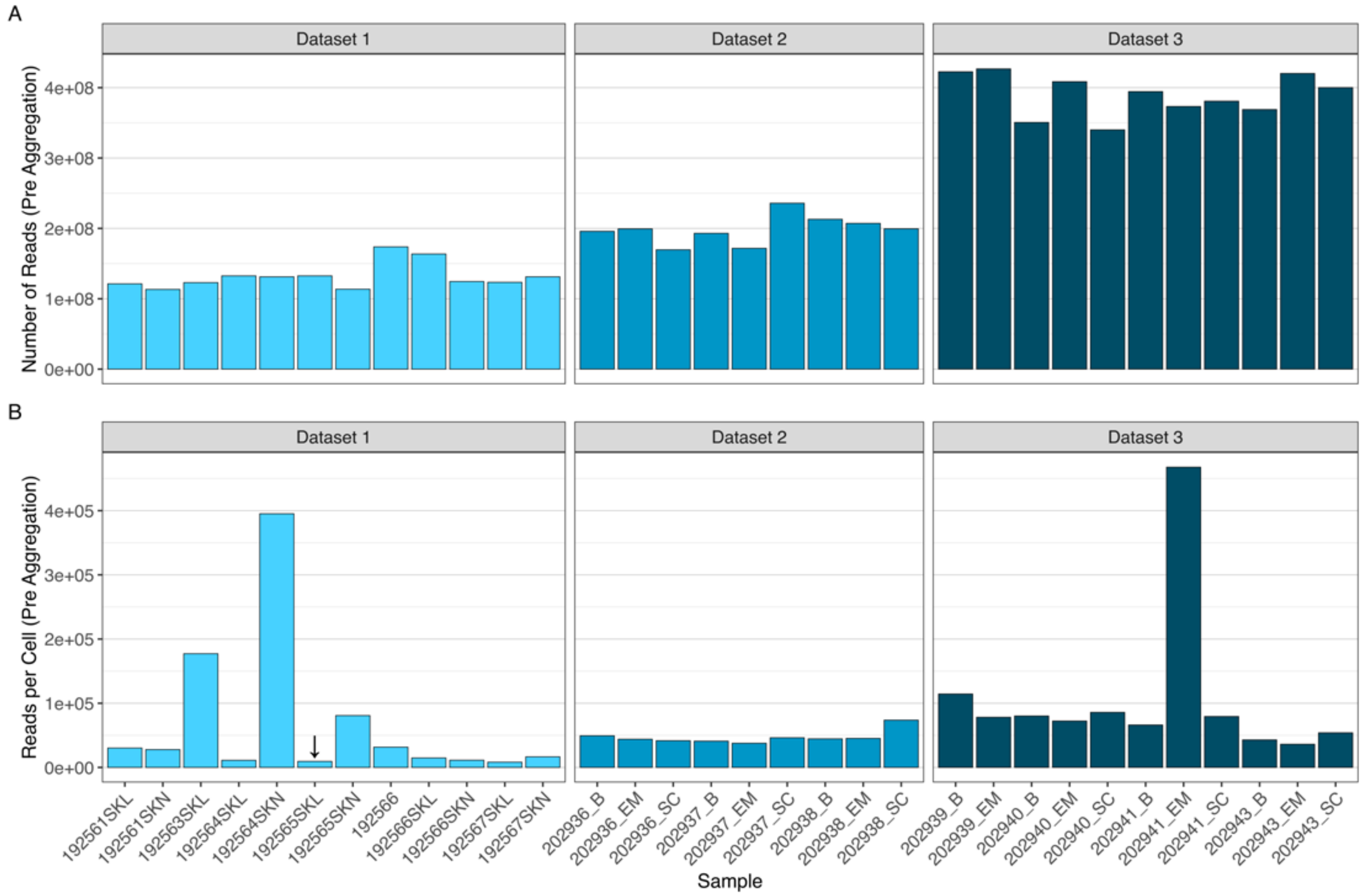

Supplemental Figure 13. Read depths prior to aggregation. A) Bar plot showing the number of reads in the hundreds of millions for each sample (blood and skin, including control and EM) prior to aggregation, grouped and colored by dataset. **B)** Bar plot showing the reads per cell for each sample, with an arrow pointing to the smallest value that was subsequently used for aggregation. Note that sample names ending in “SKL” and “_EM” represent EM samples, “SKN” and “_SC” represent control skin samples, and “_B” represent blood samples. “192566” in dataset 1 was a blood sample.

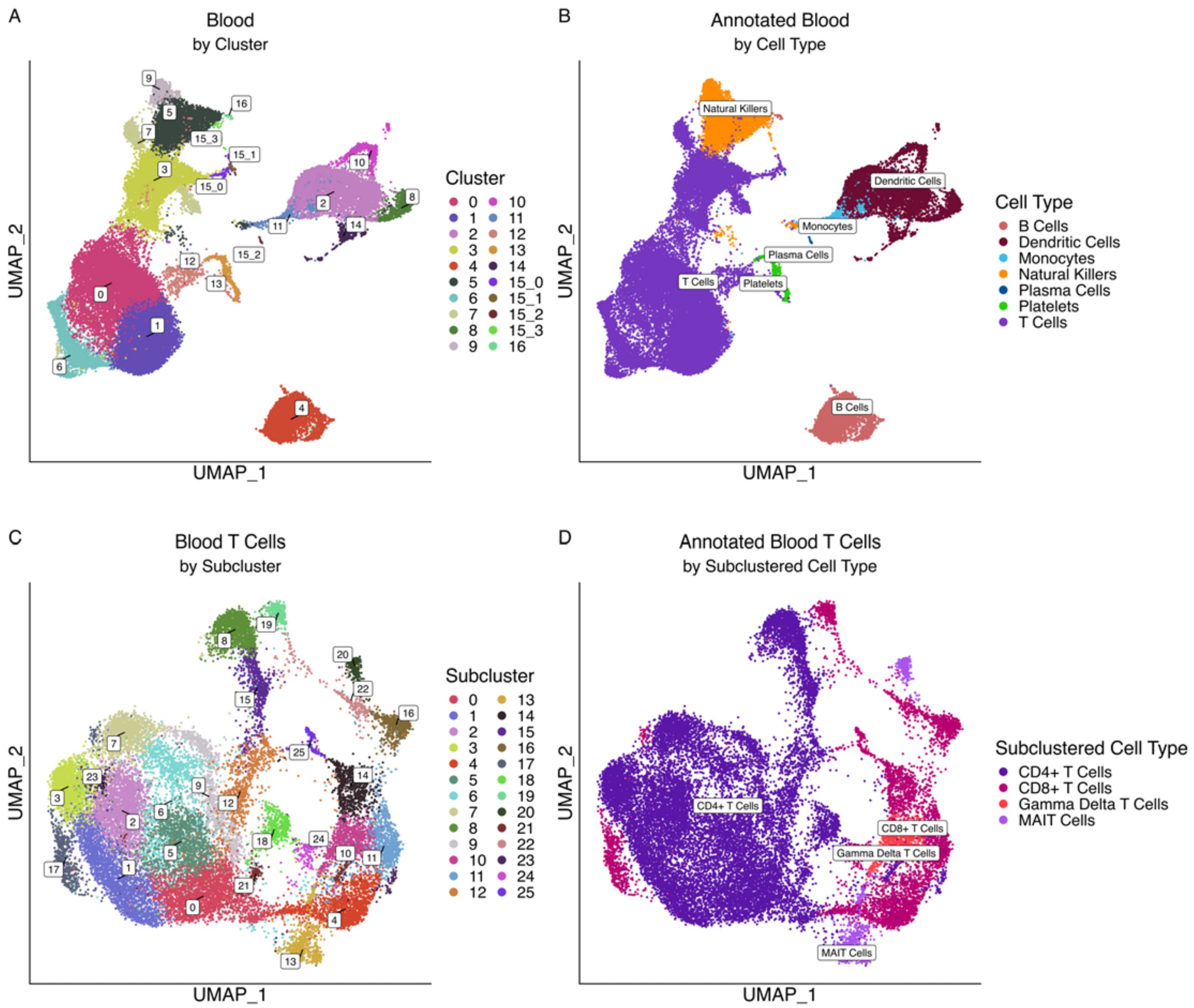

Supplemental Figure 14. Clustering and annotation of the blood cells and the blood T cells. A) UMAP showing the Seurat clusters for the blood data. Note that cluster 15 was split into four subclusters for annotation purposes. **B)** UMAP showing the annotated blood cell types. **C)** UMAP showing the Seurat clusters for the blood T cells. **D)** UMAP showing the annotated blood T cell types.
